# Beyond fear circuits: multiscale neurobiological architecture of panic disorder

**DOI:** 10.1038/s41380-026-03661-w

**Published:** 2026-05-27

**Authors:** Katharina A. Zientek, Juergen Dukart, Sarah Kreuzer, Katrin Sakreida, Mario Rastätter, Miloš Stanković, Rainer Rupprecht, Katharina Domschke, Simon B. Eickhoff, Justine Y. Hansen, Timm B. Poeppl

**Affiliations:** 1 Department of Psychiatry, Psychotherapy and Psychosomatics, Faculty of Medicine, RWTH Aachen University, Aachen, Germany; 2 Institute of Neuroscience and Medicine, Brain & Behaviour (INM-7), Research Centre Jülich, Jülich, Germany; 3 Institute of Systems Neuroscience, Medical Faculty, Heinrich Heine University Düsseldorf, Düsseldorf, Germany; 4 Department of Psychiatry and Psychotherapy, Faculty of Medicine, University of Regensburg, Regensburg, Germany; 5 Department of Psychiatry, Psychotherapy and Psychosomatics, Psychiatric Hospital, University of Zürich, Zürich, Switzerland; 6 Department of Psychiatry and Psychotherapy, Medical Center – University of Freiburg, Faculty of Medicine, University of Freiburg, Freiburg, Germany; 7 McConnell Brain Imaging Centre, Montréal Neurological Institute, McGill University, Montréal, Canada; 8 Lifespan Brain Institute, The Children’s Hospital of Philadelphia and Penn Medicine, Philadelphia, PA USA

**Keywords:** Neuroscience, Psychiatric disorders

## Abstract

Panic disorder is a prevalent and disabling condition marked by recurrent panic attacks and high treatment resistance. While previous research has focused on dysfunction within canonical fear circuits, the neurobiological basis of panic disorder may involve broader alterations across multiple brain systems. Here, we conducted a meta-analysis of functional neuroimaging experiments to identify consistent patterns of altered brain activity in panic disorder. We found increased activity in a prefrontal–hippocampus–brainstem axis, which was not confined to traditional fear-related regions. This pattern showed robust spatial associations with serotonergic and dopaminergic receptor distributions and was significantly explained by gene expression profiles of candidate genes, accounting for over one-third of the variance and supporting a polygenic model of the disorder. Further enrichment analyses revealed that the brain pattern is characterized by low neurodevelopmental and evolutionary expansion and reduced oxygen metabolism, consistent with theories of brainstem-based hypersensitivity. Functional annotation linked the identified brain pattern to emotional arousal, memory, learning, and goal-directed behavior, suggesting that panic disorder reflects psychophysiological interactions of higher-order cognitive systems and evolutionary older biological processes. These findings suggest that panic disorder involves widespread neural alterations beyond fear circuitry and highlight potential molecular and functional targets for future mechanistic and therapeutic research.

## Introduction

The term *panic* is derived from the Ancient Greek adjective πανικός (pertaining to Pan) and refers to the shepherd-god Πάν (Pan) with his goat’s feet and two horns [1]. The son of Ἑρμῆς (Hermes) was known for playing sweet and low on his pipes of reed [1], but causeless terrors were also said to come from this keen-eyed god [2]. If the φόβος Πανικός (“panic”) that fell on the Barbarians one night in their retreat during their battle with the Greeks [2] met the criteria of a *panic attack* according to the DSM-V, they must have experienced an abrupt surge of intense fear or intense discomfort that reached a peak within minutes [3], including physical (e.g., palpitations, dyspnoea, diaphoresis, chest pain, dizziness, paraesthesia, or nausea) and/or cognitive (e.g., fear, fear of losing control, or feeling of alienation) symptoms [4].

The diagnosis of *panic disorder* refers to recurrent unexpected panic attacks, followed by consistent concern about additional panic attacks or their consequences as well as significant maladaptive behavioral changes (e.g., avoidance behaviors) [3]. The considerable 1-year and lifetime prevalence of this anxiety disorder is estimated at ≈2%, has been stable over the past two decades and is higher in women than in men (≈2:1) [5–7]. First-line treatments comprise evidence-based psychotherapies and pharmacotherapy, but these are overall less effective in panic disorder than in two other highly prevalent anxiety disorders, namely social and generalized anxiety disorder [4]. More specifically, 20–40% of patients fail to respond to state-of-the-art treatment [8, 9], which may explain why the economic burden of panic disorder is highest among all anxiety disorders [10]. Given the prevalence and burden of panic disorder, it is thus remarkable that the current drug development pipeline is rather empty [4]. In this context, it has been proposed that novel therapies should address the “question of how to selectively target neuronal subpopulations that build pathological defensive responses within the fear network” [4].

However, there is no robust definition of a single neural “fear network” in the brain. Qualitative overviews suggest that perturbed threat responding in anxiety disorders involves dysfunction in circuits associated with danger responses, including the amygdala, caudate, fornix, hippocampus, insula, and stria terminalis [4]. Neuroimaging meta-analyses indicate that the rostral dorsomedial prefrontal cortex contributes to conscious threat appraisal [11], a process that in cognitive models of panic disorder is closely linked to catastrophizing and heightened interoceptive focus. While fear conditioning engages an “extended fear network” comprising the anterior insular cortex, ventral striatum and major thalamic nuclei, a large expanse of medial wall cortex, the secondary somatosensory cortex, dorsolateral prefrontal cortex, lateral premotor cortex, the ventral-posterior precuneus, lateral cerebellum, and smaller subcortical regions such as the septal-hypothalamic zone and midbrain/dorsal pons [12], it represents an associative learning mechanism that is conceptually distinct from conscious threat appraisal. Yet, the relevance of fear conditioning for panic disorder and the subjective experience of fear remains debated, as highlighted by the two-systems model [13], which distinguishes between conscious fear experiences and non-conscious defensive survival circuits, and evidence that panic patients may exhibit impaired rather than enhanced fear learning [14]. Fear generalization, which contributes to clinical anxiety, shows positive generalization, i.e., increased responding to stimuli resembling the conditioned stimulus (threat cue) in cingulo-opercular, frontoparietal, striatal-thalamic, and midbrain regions, and negative generalization, i.e., reduced responding to stimuli resembling the safety cue, in default-mode network nodes and amygdala [15]. Preliminary meta-analytic findings focused on emotion processing indicate bilateral insula/inferior frontal gyrus hyperactivation in patients with panic disorder [16]. While the “fear network“ framework has guided much of the neuroimaging literature, recent reviews of threat and safety learning [17–19] emphasize the complexity of these processes across anxiety disorders. It thus remains unclear whether panic disorder is best conceptualized as a dysfunction within this network [11, 12, 15]. In fact, no robust, disorder-specific neural signature has been established. Given the complex symptomatology of panic disorder—spanning emotional, interoceptive, cognitive, and autonomic domains—it is plausible that its neural correlates extend beyond canonical fear circuits [4]. Developmental and metabolic gradients refer to large-scale cortical axes reflecting regional variation in neurodevelopmental timing and metabolic profiles across the brain. We therefore hypothesize that panic disorder involves broader patterns of brain dysfunction, potentially encompassing phylogenetically older structures and systems involved in metabolic regulation, neurotransmission, and behavioral control.

It has been argued that anxiety disorders may be neurodevelopmental in their origins and that genetic and epigenetics influences on brain development and function are crucial to the pathophysiology [20, 21]. This perspective aligns with research on endophenotypes in anxiety disorders [22–25], which highlights intermediate phenotypes bridging genetic risk and clinical expression. Heritability estimates converge to rates of around 50% for panic disorder [26]. Yet, genetic and epigenetic testing cannot be recommended for clinical practice, given the limited evidence of specific risk genes [4]. However, respective studies might provide mechanistic insights and hence avenues for new treatments [4]. In particular, studying the genetic basis of putative functional brain abnormalities and, in turn, association of the latter with behavioral abnormalities could link pathophysiology to psychopathology. Unfortunately, the exact relationship between genetic mechanisms, brain development, functional brain alterations and specific symptoms of panic disorder remains unclear.

Here, we aim to characterize the neurobiological architecture of panic disorder by integrating large-scale neuroimaging evidence with molecular and behavioral annotations. Specifically, we ask whether consistent patterns of altered brain activity in panic disorder show systematic spatial associations with key biological features, including neurotransmitter systems, gene expression profiles, developmental and metabolic gradients, and functional domains of cognition and emotion. To address this, we first conduct a meta-analysis of functional neuroimaging studies to identify the pattern of abnormal brain activity in patients with panic disorder. Based on prior evidence implicating neurotransmitter systems in panic disorder, we expect to find a specific chemoarchitectonic signature of disorder-related brain activity. We further explore whether expression of candidate genes previously linked to panic disorder explains variance in this brain pattern, and whether the pattern preferentially localized to phylogenetically older brain regions with lower metabolic rates. Finally, we perform an unbiased, data-driven characterization to determine which mental processes and behavioral domains are most strongly associated with this brain pattern using meta-analytic functional annotations. By combining neuroimaging meta-analysis with enrichment analyses across multiple biological scales, this work seeks to provide an integrative perspective on the pathophysiology of panic disorder. This integrative approach aims to move beyond narrow circuit models and provide a more comprehensive view of the neurobiology of panic disorder.

## Methods

### Data selection

Our study was conducted in accordance with the “Preferred Reporting Items for Systematic Reviews and Meta-Analyses (PRISMA)” statement and best practice guidelines for neuroimaging meta-analysis [27, 28].

A principled procedure to identify the relevant experimental studies published from January 1, 1997, to March 31, 2025, was used (Supplementary Fig. 1). First, we selected studies through a standard search in the PubMed (https://www.ncbi.nlm.nih.gov/pubmed/), ISI Web of Science (https://www.webofknowledge.com) and Scopus (https://www.scopus.com/) databases using the term “panic disorder” in combination with “fMRI”, “functional MRI”, “functional magnetic resonance”, “PET”, “positron emission”, “ASL”, “arterial spin labeling”, “MEG”, “magnetoencephalography”, “neuroimaging”, or “imaging.” Second, further studies were found by means of the “related articles” function of the PubMed database and by tracing the references from the identified papers and review articles. Task-based neuroimaging experiments were considered relevant when they reported either (1) group comparisons between patients with panic disorder and controls, (2) correlations of brain activity with measures of symptom severity. Only experiments reporting results of whole-brain group analyses with coordinates referring to a standard reference space (Talairach-Tournoux or Montreal Neurological Institute (MNI)) were included. Results of region-of-interest or seed-based connectivity analyses and studies not reporting stereotaxic coordinates were excluded. We did not apply an external statistical significance threshold for including activation or deactivation foci. Because statistical criteria and correction methods vary widely across studies, imposing a uniform threshold would risk systematic selection bias. Instead, we included all whole-brain, peer-reviewed experiments, assuming that reported coordinates met each study’s methodological standards. The ALE random-effects framework then evaluates convergence across studies, reducing the influence of individual thresholding choices.

Study eligibility was independently assessed by two reviewers (K.Z. and T.B.P.). In cases of uncertainty or disagreement regarding inclusion or exclusion of a study, a senior expert (S.B.E.) was consulted to resolve discrepancies and ensure consistency with predefined criteria.

On the basis of these search criteria, 34 papers were found to be eligible for inclusion into the meta-analyses (Supplementary Table 1). Thirty-two functional magnetic resonance imaging and two positron emission tomography but no magnetoencephalography studies or arterial spin labeling studies fulfilled our search criteria. Together, these papers reported 620 foci obtained from 106 experimental contrasts (Supplementary Table 2).

The count of these foci was composed of 315 activations from 54 direct group comparisons (‘panic disorder > controls’) and 11 foci of positive correlations between brain activity and symptom severity from 2 analyses as well as 224 deactivations from 38 direct group comparisons (‘panic disorder < controls’) and 1 focus of negative correlation between brain activity and symptom severity from 1 analysis. In addition, 11 analyses on main group or interaction effects reported altogether 69 foci indicating differential activity between patients with panic disorder and controls. Differences in coordinate spaces (Talairach vs. MNI space) were accounted for by transforming coordinates reported in Talairach space into MNI coordinates using a linear transformation [29]. Because participant overlap across publications is rarely reported in primary studies, sample independence was evaluated based on available information (e.g., sample size, demographics, and recruitment descriptions), which provides an approximate but field‑standard approach (see Supplementary Table 1).

**Fig. 1:**
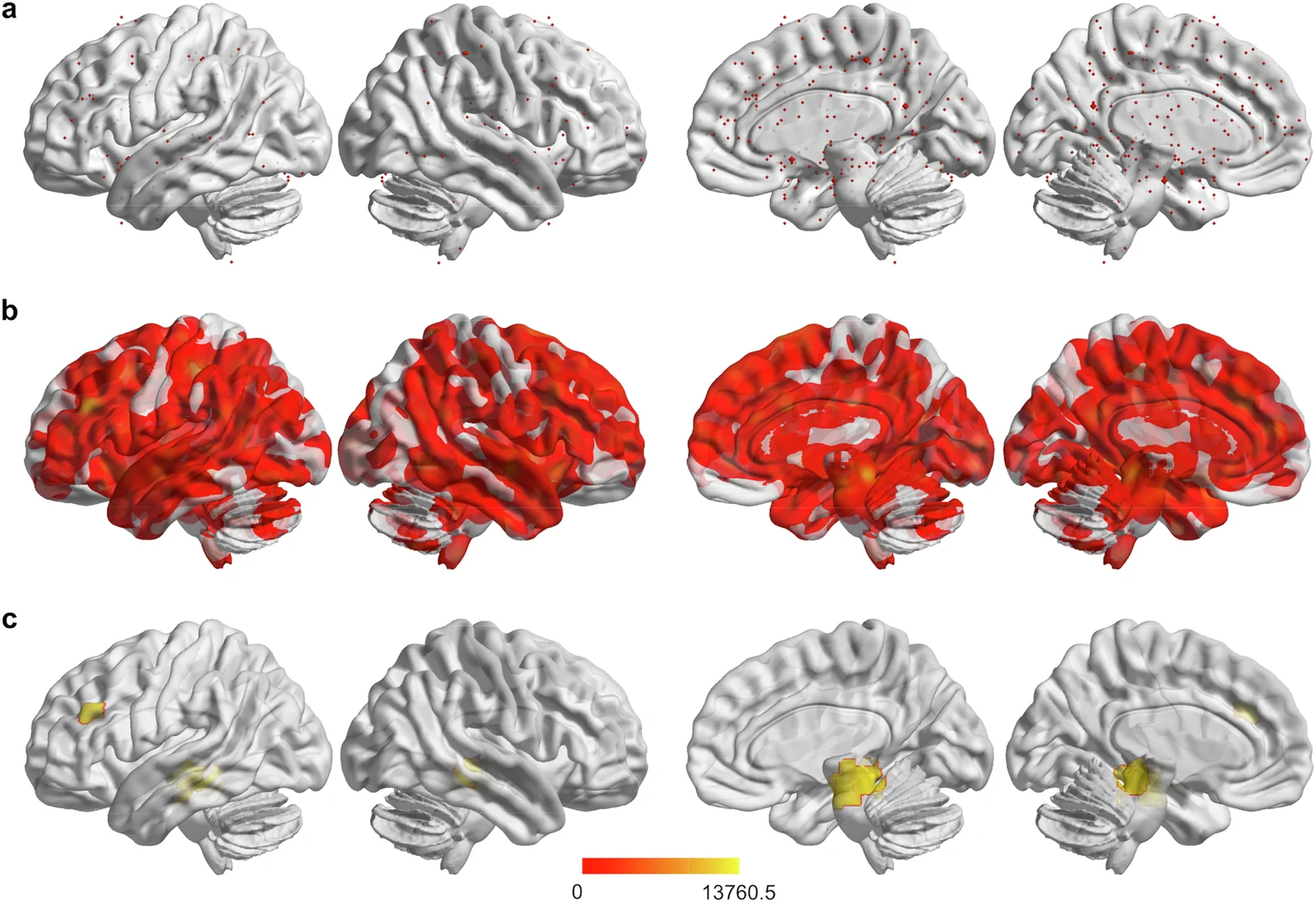
Brain regions showing increased brain activity associated with panic disorder. **a**, The meta-analysis included 326 foci of increased brain activity in patients with panic disorder from 56 experiments. **b**, For each voxel, activation likelihood estimates (ALE) reflect the union of the modeled activation (MA) maps across all experiments included. **c**, Nonparametric tests revealed four clusters of significant convergence of altered brain activity in corresponding experiments (*p* < 0.05, corrected). These regions comprised the left dorsolateral prefrontal cortex, left hippocampus, and brainstem (cf., Table 1). The color bar indicates TFCE-values. L, left; TFCE, threshold-free cluster enhancement.

First, convergence of all reported foci was analyzed for the main effect of aberrant brain activity in panic disorder (106 experimental contrasts, 620 foci; see Supplementary Figs. 2 and 3). Furthermore, we assessed convergence of reported activation foci indicating increased brain activity in panic disorder by pooling direct comparisons and correlational analyses (56 experimental contrasts, 326 foci; Fig. 1a/b). In an analogous manner (i.e., by pooling group comparisons and correlational analyses), we tested for convergence of reported deactivation foci (39 experimental contrasts, 225 foci). The denoted sample sizes (i.e., number of experimental contrasts) have been shown to be sufficient to achieve robust meta-analytic estimates [30].

### Activation likelihood estimation

All statistical analyses were carried out using the revised ALE algorithm for coordinate-based meta-analysis of neuroimaging results (Supplementary Methods) [31, 32]. This algorithm aims to identify regions with a convergence of reported coordinates across experiments that is higher than expected from a random spatial distribution. Foci are treated as centers of 3D Gaussian probability distributions capturing the spatial uncertainty associated with each focus [31]. Here, the between-subject variance is weighted by the number of participants per study, since larger sample sizes should provide more reliable approximations of the “true” activation effect and should therefore be modeled by more “narrow” Gaussian distributions. Subsequently, probabilities of all foci reported of a given experiment were combined for each voxel, yielding a modeled activation (MA) map [32]. To avoid bias from multiple contrasts within the same study (“double dipping”), we organized foci by subject group. This ensures that multiple foci from a single experiment do not artificially increase MA values, and that contrasts from the same dataset do not cumulatively inflate ALE scores [32]. It can thus be ruled out that effects are amplified by nonorthogonal contrasts (i.e., from the same study) being submitted to the same analysis. In other words, each study contributes only once per subject group, preventing amplification of effects from non-independent contrasts. The union across these individual MA maps was then calculated to obtain voxel-wise ALE scores, i.e., an ALE map, which quantified the convergence across experiments at each location in the brain. More precisely, ALE scores were calculated by taking the voxel-wise union of their probability values (i.e., $1-\,{\Pi }_{i}^{{N}_{{foci}}}(1-p\left(i\right))$, where $p\left(i\right)$ is the probability associated with the *i*th focus at this particular voxel). To distinguish “true” from random convergence, ALE scores (i.e., the ALE map) were compared to an empirical null distribution of random spatial associations between experiments using a non-linear histogram integration algorithm [31]. The resulting random-effects inference focuses on the above chance convergence across studies rather than the clustering within a particular study [33]. This null hypothesis was derived by computing the distribution that would be obtained when sampling a voxel at random from each of the MA maps and taking the union of these values in the same manner as for the (spatially contingent) voxels in the original analysis [31]. The *p*-value of a “true” ALE score was then given by the proportion of equal or higher values obtained under the null distribution. The resulting nonparametric *p*-values were then assessed employing threshold-free cluster enhancement using 10,000 permutations for correcting multiple comparisons [31].

Contributions were estimated by determining for each included experiment, how much it contributes to the summarized test-value (i.e., the ALE score) of a specific cluster. This was done by computing the ratio of the summarized test-values of all voxels of a specific cluster with and without the experiment in question, thus estimating how much the summarized test-value of this cluster would decrease when removing the experiment in question [27].

For anatomical labeling, we capitalized on cytoarchitectonic maps of the human brain provided by the Statistical Parametric Mapping (SPM) Anatomy Toolbox (JuBrain Anatomy Toolbox v3.0) [34–36]. Clusters were thus assigned to the most probable histologically defined area at the respective location. This probabilistic histology-based anatomical labeling is reported in the results tables. References to details regarding cytoarchitecture are given in the table notes. For areas not listed in this tool, we relied on the Talairach Daemon [37], including Structural Probability Maps, which in turn are based on 50 or more MRI brain volumes that were automatically labeled using the non-linear image-matching ANIMAL algorithm [38].

### Spatial correlation with receptor/transporter densities

Finally, we evaluated the topographical relationship between altered brain activity in panic disorder and the distribution of receptor/transporter systems as derived from positron emission tomography (PET) studies in independent healthy volunteers. To this end, we used Spearman’s rank partial correlation implemented in the JuSpace toolbox [39], controlling for partial volume effects and spatial auto-correlation. Receptor maps included cholinergic, dopaminergic, GABAergic, glutamatergic, noradrenergic, opioidergic, and serotonergic systems (Supplementary Table 3). For these analyses, we used the unthresholded whole-brain meta-analytic maps, which preserve the continuous spatial pattern of cross-study convergence. These maps were parcellated together with all neurotransmitter maps using the 119-region Neuromorphometrics atlas provided with JuSpace (https://github.com/juryxy/JuSpace/tree/JuSpace_v1.5/JuSpace_v1.5/atlas), and correlations were computed across all parcels. This whole-brain parcellation avoids biases that would arise from restricting analyses to thresholded ALE clusters. Receptor maps showing significant associations (*p* < 0.05, FWE rate corrected) were subsequently entered into multiple linear regression models to assess the specificity of their contributions.

### Gene expression analysis

To test whether the significant associations observed in the above analysis are also found at the level of transcriptomics, we downloaded the interpolated mRNA data in MNI space (https://www.meduniwien.ac.at/neuroimaging/mRNA.html) for genes encoding the respective receptors as derived from the Allen Human Brain Atlas [40]. We also used this database to investigate whether the meta-analytic brain map of increased activity in panic disorder is linked to established candidate genes in panic disorder [41–43]. The final selection of 13 genes was based on candidate genes as derived from our receptor/transporter association analysis or as specified by a meta-analysis, an up-to-date chapter in the New Oxford Textbook of Psychiatry, or an authoritative review [41–43]. In this analysis, parcel-wise unthresholded TFCE values served as the dependent variable, representing the spatial pattern of increased activity in panic disorder. Parcel-wise mRNA expression levels of all 13 selected genes were used as predictors to assess whether their combined spatial distribution relates to the meta-analytic activation pattern. To follow the concept of the polygenic risk score (PRS), we employed multiple linear regression models entering all mRNA data into a single model and comparing the overall explained variance to the one obtained using permuted mRNA data adjusting for spatial autocorrelation.

**Fig. 2:**
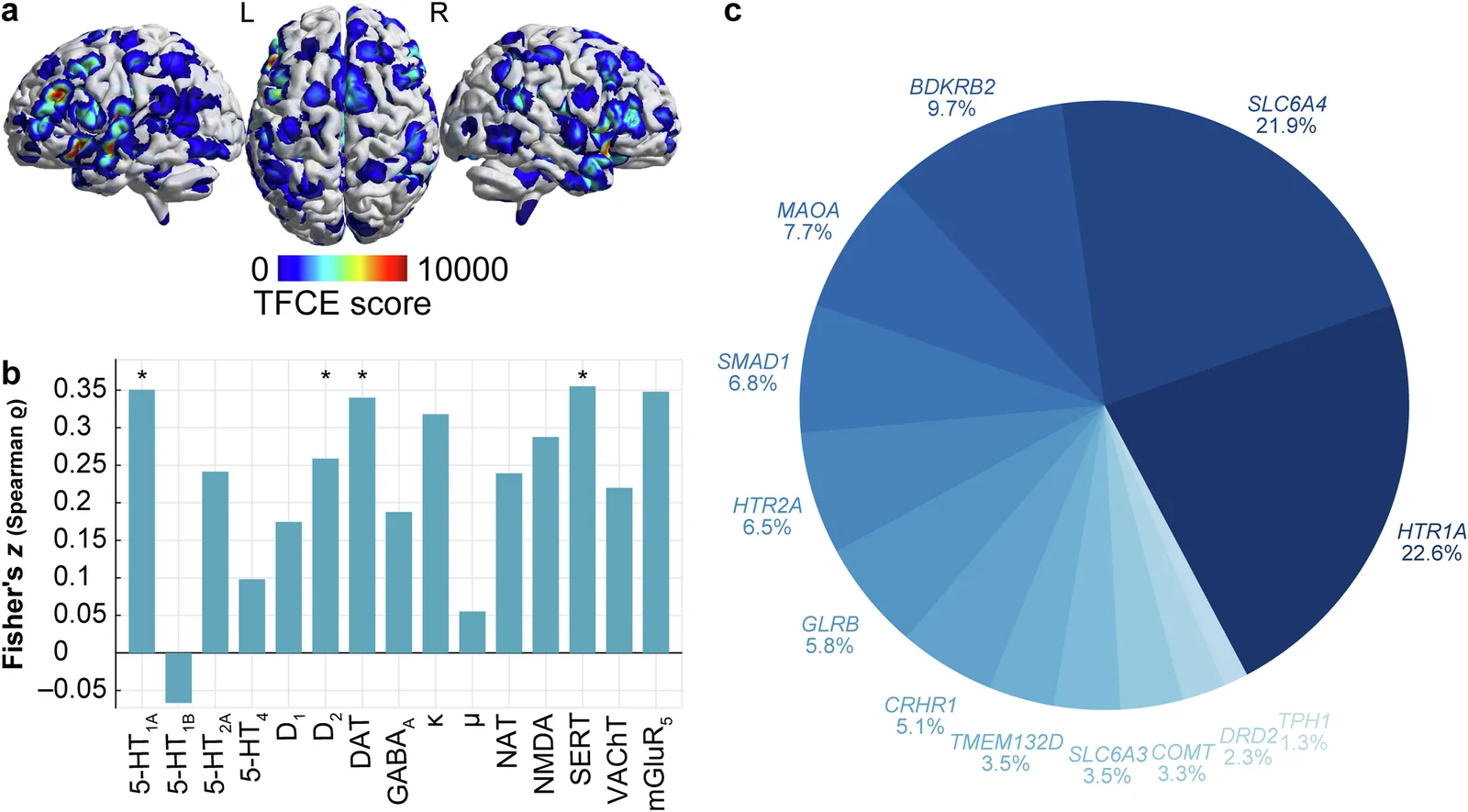
Relationship of altered brain activity in panic disorder with chemoarchitecture and gene expression. **a, b**, Meta-analytically derived increased brain activity (**a**) was spatially related to distribution of 5-HT_1A_, SERT, D_2_, and DAT (* *p* < 0.05, family-wise error corrected) (**b**). The color bar represents *TFCE*-values as weighted sums of the entire local clustered signal on the unthresholded brain map. A multiple linear regression model was fit between expression profiles of candidate genes in panic disorder and the meta-analytic brain pattern), indicating that all genes had explanatory power (*R*^2^ = 0.341). Dominance analysis was applied to the independent variables (mRNA expression data) to determine which genes were contributing most to the model fit. Percent contribution is shown in the pie char (**c**).

Next, we employed dominance analysis to determine the relative contribution (“dominance”) of each independent variable to the overall fit (*R*^2^) of the multiple linear regression model (https://github.com/dominance-analysis/dominance-analysis [44]). To this end, the same regression model was fitted on every combination of input variables (i.e., 2^*p*^ − 1 submodels for a model with *p* input variables). Total dominance is defined as the average of the relative increase in *R*^2^ when adding a single input variable of interest to a submodel, across all 2^*p*^ − 1 submodels. Since the sum of the dominance of all input variables is equal to the total *R*^2^ of the complete model, total dominance is an intuitive method that partitions the total effect size across predictors. Therefore, unlike other methods of assessing predictor relevance, such as methods based on regression coefficients or univariate correlations, dominance analysis accounts for predictor-predictor interactions and is easily interpretable. Dominance was normalized by the total fit (*R*^2^) of the model, to make dominance fully comparable both within and across models. The normalized total dominance (percent contribution) is illustrated in the pie char in Fig. 2.

### Expansion and metabolism

To investigate how the brain map of altered brain activity in panic disorder relates to brain development and metabolism, we used the neuromaps package (https://github.com/netneurolab/neuromaps) [45], which allows for sharing, transforming, and comparing brain maps. This package contains curated reference brain maps from the literature that have been published to facilitate contextualization of brain annotations. We used the neuromaps transformation functions to convert the meta-analytic brain map of panic disorder (“source map”) to the cortex space of three selected normative brain maps from neuromaps (“target maps”): (1) brain map of developmental expansion, derived from healthy infants and adults [46], where developmental expansion describes the degree to which cortical regions increase in surface area from early development into adulthood; (2) (sample size weighted) brain map of evolutionary expansion, representing the ratio of the surface area in humans to that of chimpanzees [47] or macaques [48]; (3) brain map of oxygen metabolism, expressed as cerebral metabolic rate for oxygen (CMRO_2_) [49]. Outliers were quantified using the commonly used 1.5 × interquartile range (IQR) rule and accounted for by winsorizing. We relied on the default, primary map comparison workflow, which uses the standard Pearson correlation to test the association between provided maps. For significance testing, we implemented non-parametric, spatial autocorrelation-preserving null models for statistical comparison between the source and the target brain maps [50]. Multiple comparison adjustments were conducted to control the FWE rate.

### Behavioral profiling

To obtain a data-driven functional characterization of the spatial pattern of meta-analytically derived altered brain activity in panic disorder, we capitalized on Neurosynth [51], one of the largest extant repositories of functional brain-imaging results with rich experimental descriptions. We obtained unthresholded voxel-wise probabilistic measures of the association between our meta-analytic maps and terms of neurocognitive processes from Neurosynth. This meta-analytic tool synthesizes results from more than 14,000 published functional MRI studies by searching for high-frequency key words (such as “pain” and “attention”) that are published alongside standardized coordinates of neural activity responses. Neurosynth measures associations as the probability that a given term is reported in a brain-imaging experiment if neural activity changes are observed at a given brain location (the association test). The approach is based on the co-occurrence that certain brain areas are frequently mentioned in conjunction with certain words. Behavioral term associations were derived from large‑scale reverse‑inference models that adjust for base‑rate term frequencies across the neuroimaging literature, yielding standardized estimates of the functional domains most strongly associated with the observed activation pattern. Neurosynth matches voxels to more than 1000 terms, many of which are anatomical (“accumbens”, “dorsolateral”), disease-specific (“adhd”, “ad”), or otherwise non-psychological in nature and difficult to interpret (“adult”, “arterial spin”). Therefore, to reduce the list of terms to those that involve psychological processes, and following the methodology used originally by Hansen et al. [52], we used the Cognitive Atlas [53], a public ontology of cognitive science. This resulted in 125 terms in the intersection of the Cognitive Atlas and Neurosynth, ranging from umbrella terms (e.g., “attention”, “emotion”) to specific terms of cognitive processes (e.g., “visual attention”, “episodic memory”), types of behavior (e.g., “eating”, “sleep”), and emotional states (e.g., “fear”, “anxiety”). Note that these features were fetched programmatically, using the neurosynth Python package (https://github.com/neurosynth/neurosynth). For each term, we correlate the Neurosynth-derived meta-analytic volumetric map to the brain-wide ALE values (Pearson correlation). These volumetric correlations are done using the neuromaps Python package (https://github.com/netneurolab/neuromaps), which stacks brain voxels (masking background or other non-brain voxels) into a vector before correlating the two vectorized volumetric images [45]. Correlations highlight the spatial overlap between functional activations associated with a given cognitive term and the obtained whole-brain correlates of panic disorder.

## Results

### Coordinate-based meta-analysis

We used activation likelihood estimation (ALE) meta-analysis of pertinent neuroimaging data from ≈1600 patients and controls to generate a brain map of panic disorder. This brain map is based on experiments employing various tasks and reporting group comparisons between patients with panic disorder and controls or correlations of brain activity with measures of symptom severity. Across 106 experiments, no convergence of *generally altered* brain activity in panic disorder was observed. In contrast, convergence of *increased* brain activity in patients with panic disorder across 56 experiments was located in the left dorsolateral prefrontal cortex, left hippocampus, and several brainstem nuclei (Fig. 1c and Table 1), consistent with the notion that abnormal anxiety is reflected by neural hyperactivity, but without evidence for a role of the amygdala in the pathophysiology of panic disorder [54]. Local maxima of convergence within the brainstem were identified in periaqueductal gray, inferior colliculus, pedunculotegmental nucleus, microcellular tegmental nucleus, medial parabrachial nucleus, substantia nigra, and ventral tegmental area (Table 1). Although some theoretical accounts have proposed that pathological anxiety may involve deficits in neural inhibitory control networks, our follow-up analyses indicating no convergence of *generally decreased* neural activity associated with panic disorder provided no evidence to support the claim that abnormal anxiety is linked to such inhibitory deficits [55].

**Table 1:** Increased brain activity in panic disorder.

Macroanatomical Location	Cluster Size in Voxels	MNI Coordinates			TFCE Score
		x	y	z	
L Periaqueductal gray, inferior colliculus, pedunculotegmental ncl.	185	−10	−34	−12	13760.5
R Microcellular tegmental nucleus		10	−32	−10	12439.4
R Periaqueductal gray, Inferior colliculus		8	−34	−12	12428.8
R Medial parabrachial nucleus		6	−34	−22	12048.1
L Substantia nigra	77	−12	−20	−16	12113.8
L Ventral tegmental area – parabrachial pigmented nucleus complex		−4	−22	−14	12019.0
L Substantia nigra		−10	−14	−22	11758.8
L Hippocampus (dentate gyrus)	53	−28	−24	−10	12145.5
L Hippocampus		−22	−24	−6	11800.4
L Hippocampus		−22	−22	−6	11800.4
L Dorsolateral prefrontal cortex	9	−48	32	28	11870.6

There was no convergence of generally aberrant brain activity across 106 experiments featuring 620 foci or of decreased brain activity across 39 experiments featuring 225 foci. Across 56 experiments featuring 326 foci of increased activity related to panic disorder, ALE revealed four clusters of significant convergence. Results are corrected for multiple comparisons using threshold−free cluster enhancement (*p* < 0.05).

*ALE* activation likelihood estimation, *MNI* Montreal Neurological Institute.

Given that basic neuroscience research suggests that psychopathology in anxiety disorders involves dysfunction in brain circuitries that support core psychological processes, in particular emotion and learning [4], we asked whether the significant findings (i.e., increases in brain activity) might be driven by corresponding experimental paradigms (cf. Supplementary Table 2). To test whether our findings were driven by specific task types, we performed contribution analyses and additional sensitivity checks. All major paradigms contributed to the observed clusters, except for the small dorsolateral prefrontal cortex cluster, which was only supported by tasks involving emotional stimuli. Statistical comparisons between meta-analytic maps for different task categories (emotional vs. non-emotional; learning vs. non-learning all experiments) revealed no significant differences (Supplementary Figs. 4–7). Furthermore, non-parametric, spatial autocorrelation-preserving null models confirmed that maps derived from different paradigms were highly similar to the overall map (all r ≥ 0.562, all p_spin-FWE_ < 0.001) as well as to each other (all r ≥ 0.230, all p_spin-FWE_ ≤ 0.042). Also narrowing the meta-analyses to MRI experiments led to the same results. These results indicate that the identified brain pattern reflects a general feature of panic disorder rather than being driven by a specific task. The pattern observed here contrasts with findings in other major psychiatric disorders such as schizophrenia, mood disorders, or personality disorders, which typically show reduced rather than increased activation in comparable analyses [55–59], although formally quantifying disorder-specificity would require direct map-to-map comparisons with large-scale meta-analytic brain maps from those conditions. To nevertheless provide a quantitative benchmark, a spatial dissimilarity analysis comparing the panic disorder map with a large transdiagnostic ALE map [55] showed marked and, at intermediate thresholds, significant dissimilarity (e.g., D = 0.995, p = 0.026 at the 60th percentile, 69 parcels), indicating that the observed pattern reflects largely panic‑specific topography.

### Relationship to molecular architecture

Given that the increased brain activity localizes to a prefrontal-hippocampus-brainstem axis, we sought to identify the relationship between the brain map of panic disorder and neurotransmitter systems. We used data from a recent PET atlas of nine neurotransmitter systems in the human brain to estimate distributions of 15 neurotransmitter receptors and transporters [45, 60]. Correlational analyses between the brain map of increased neural activity in panic disorder and density of neurotransmitter receptors/transporters in the human brain revealed a significant positive relationship (receptor: mean Fisher’s *z*, *p*-value) to serotonergic (serotonin 1A (5-HT_1A_): r = 0.351, p_FWE_ = 0.040; serotonin transporter (SERT): r = 0.355, p_FWE_ = 0.014) and dopaminergic (D_2_: r = 0.259, p_FWE_ = 0.045; dopamine transporter (DAT): r = 0.340, p_FWE_ = 0.014) systems (Fig. 2a/b). That is, the higher the availability of respective receptors in a given brain region as derived from healthy volunteer studies, the more increased the brain activity within this brain region among patients with panic disorder. The associations with 5-HT_1A_ (p = 0.003) and DAT (p = 0.003) remained significant when testing for specificity of the respective findings in a multiple linear regression including all of the above neurotransmitter maps. As an additional methodological check, we examined whether the spatial pattern of panic‑related hyperactivation aligns with canonical macroscale anatomical gradients. Correlations with both the principal functional connectivity gradient and the T1w/T2w myelin map were negligible (|r| <0.02, *p* > 0.88), indicating that these gradients do not account for the observed spatial pattern. Taken together, we identified a robust relationship of increased brain activity in panic disorder with serotonergic and dopaminergic systems, with the most specific link to 5-HT_1A_ receptor distribution and dopamine transporter availability.

### Relationship to gene expression

Next, we asked whether the brain map of panic disorder might be determined by genetic influence. We selected 13 established candidate genes in panic disorder and obtained the distribution of their expression across the brain from the Allen Human Brain Atlas [61]. We computed multiple linear regressions fitting all Allen Human Brain Atlas mRNA data [61] relating to the significant receptor/transporter findings as well as mRNA data of established candidate genes in panic disorder into a single model. Next, we applied dominance analysis to estimate the relative contribution (“dominance”) of each gene to the overall fit (*R*^2^) of the model (Fig. 2c). The overall-permutation-based *p*-value indicated that the model was significant (*R*^2^ = 0.341; p = 0.008) and hence that expression of at least one of these genes had explanatory power. Analysis of the individual contributions showed that all genes significantly contributed to explain the pattern of altered brain activity in panic disorder. Three serotonin system-related genes encoding 5-HT_1A_ (*HTR1A*), SERT (*SLC6A4*) and monoamine oxidase A (*MAOA*) emerged among the top 5 dominant genes and, together with *HTR2A*, accounted for 58.7% of the overall fit. In contrast, the dopaminergic system, i. e., activity of *DRD2* encoding the dopamine receptor D_2_, *COMT*, encoding the catechol-*O*-methyltransferase enzyme, and *SLC6A3* encoding the dopamine transporter made the least dominant contribution (9.1% combined). Microarray expression of six further candidate genes implicated in panic disorder also mapped to the meta-analytic source map: *BDKRB2*, encoding the bradykinin receptor B_2_; *CRHR1*, encoding the corticotropin-releasing hormone receptor 1; *GLRB*, encoding the glycine receptor; *SMAD1*, encoding the SMAD family member 1 protein; and *TMEM132D*, encoding the homonymous single-pass transmembrane protein; with *BDKRB2* as the third most dominant among all genes.

### Relationship to developmental and metabolic features

**Fig. 3:**
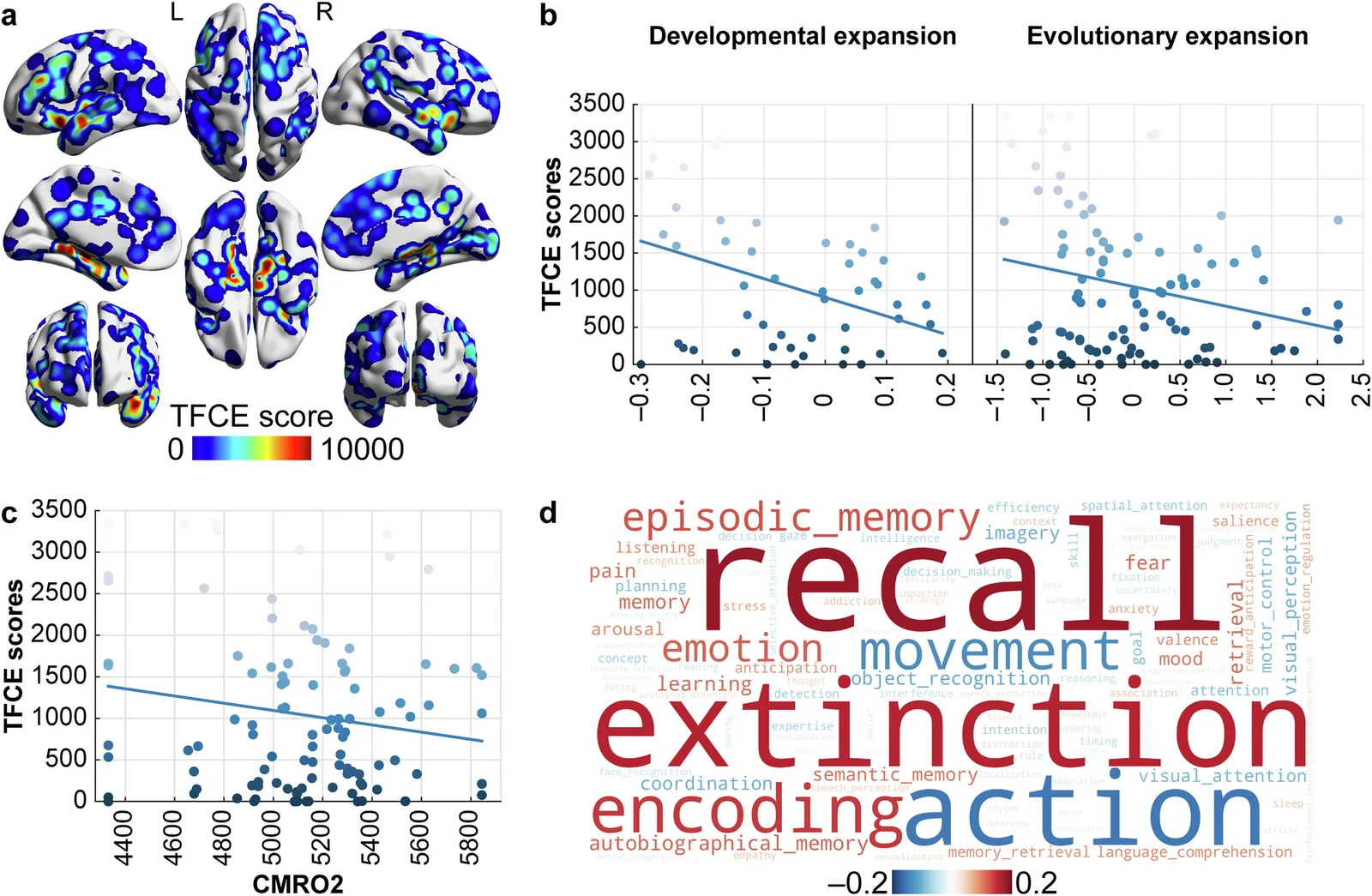
Relationship of altered brain activity in panic disorder with brain expansion, brain metabolism, and behavior. **a, b**, Meta-analytically derived panic disorder-related brain activity (**a**) was spatially related to (**b**) developmental and evolutionary brain expansion. **c**, Contextualizing this source map with respect to a normative map of oxygen metabolism, we found that areas with the greatest disorder-related increase in activity feature the lowest metabolic rate of oxygen. **d**, Querying the Neurosynth database delinated significant similarities between a given ontological term’s functional activity patterns and the meta-analytically derived increased brain activity maps of panic disorder (red/blue = positive/negative correlation, range = [−0.2, 0.2]). Word size represents relative magnitude of a given significant association.

The “suffocation false alarm” theory of panic disorder has conceived unexpected panic attacks as a primal defensive reaction to threat within the internal milieu of the body that is mediated by phylogenetically older brain structures, which process basic information related to the body’s internal milieu [62, 63]. Next, we therefore used spatial null models to contextualize the brain map of altered brain activity in panic disorder to developmental and metabolic features. That is, we correlated the meta-analytic map with target maps of brain expansion and metabolism and tested the significance using a spatial autocorrelation-preserving null model (‘spin test’). We found that panic disorder-related brain activity is enriched in regions with least neurodevelopmental (r = −0.439, p_spin–FWE_ = 0.013) and evolutionary expansion (r = −0.257, p_spin–FWE_ < 0.001) (Fig. 3a/b). This is consistent with the notion that panic symptoms originate from the phylogenetically older brain structures. The “suffocation false alarm” theory explicitly supposes a suffocation monitor to misfire an evolved suffocation alarm system, considering carbon dioxide hypersensitivity as a result of the deranged suffocation alarm monitor [62]. We therefore ask whether the brain map of panic disorder relates to the metabolic rate of oxygen, given that an integrated suffocation alarm needs to deal with both carbon dioxide and oxygen. We also find that the map is negatively correlated with cerebral oxygen metabolism, which suggests that function is disturbed in regions featuring greater metabolic resistance and efficiency—that is, regions characterized by lower baseline oxygen consumption and thus greater energetic efficiency—which may thus be well suitable as gauges of the internal milieu (r = −0.171, p_spin–FWE_ = 0.021) (Fig. 3c).

### Relationship to behavior

To establish a relationship between increased brain activity in patients with panic disorder and mental processes, we used the Neurosynth meta-analytic database for probabilistic measures that specific terms (e.g., “attention”, “emotion”, or “sleep”) are functionally related to specific brain regions [51]. This quantity mirrors how often specific terms and voxel coordinates are published in conjunction with one another. We assessed the correlation of panic disorder-related brain patterns with the individual term maps from this database. Across the pattern of increased brain activity, functional annotations from Neurosynth with large positive loadings were significantly related to memory and cognition (e.g., “memory”, “encoding”, “retrieval”), emotion (e.g., “arousal”, “emotion”, “fear”, “mood”), and learning (e.g., “extinction”, “learning”). In contrast, terms with large negative loadings related to motor action (e.g., “action”, “movement”, “motor control”), attentional processes (e.g., “object recognition”, “spatial attention”, “visual perception”), and goal-directed behavior (e.g., “coordination”, “decision making”, “planning” (Fig. 3d).

## Discussion

Our meta-analyses revealed a consistent pattern of *increased* activity in the dorsolateral prefrontal cortex, anterior hippocampus, and several brainstem nuclei including periaqueductal gray and substantia nigra in patients with panic disorder. These regions are not part of the mid dorsomedial prefrontal cortex/dorsal anterior cingulate cortex “core” fear network described by Mechias and colleagues, which is “activated irrespective of how fear was learnt” [11]. Instead, they overlap in part with structures engaged during human fear conditioning [11, 12]. While dorsolateral prefrontal cortex and brainstem nuclei have been assigned to a “central autonomic-interoceptive network” within this neural framework, the hippocampus is considered to route “safety signal” (i.e., non-threat) processing [12]. In accordance with this notion, a recent meta-analysis demonstrated that the dorsolateral prefrontal cortex and brainstem nuclei are also crucial for fear extinction learning, while the hippocampus is specifically involved in fear extinction recall [64]. These findings remarkably match recent meta-analytic results indicating that left dorsolateral prefrontal cortex and brainstem nuclei positively code conditioned fear generalization, while negative generalization effects are located in the anterior hippocampus [15]. According to the corresponding neural model of fear generalization, stimulus information travels on a “high road” via sensory cortices as well as on a quick and dirty “low road” via the amygdala to the hippocampus, which assesses the overlap of the stimulus with previously encoded threat stimuli by schematic matching [15]. Pattern separation is followed by activation of the fear inhibiting ventromedial prefrontal cortex, whereas pattern completion is followed by activation of threat excitatory regions including brainstem nuclei [15]. These threat-related activations next engage control areas of the brain, such as the dorsolateral prefrontal cortex, which deploy attentional and emotion-regulation processes for response optimization [15]. That our meta-analysis revealed increased activity in the left anterior hippocampus, several brainstem nuclei, and left dorsolateral prefrontal cortex might indicate that panic attacks result from dysfunction in neural circuits of memory-related stimulus pattern evaluation, threat-related processes, and emotiocognitive control, suggesting that panic disorder involves alterations across multiple functional domains rather than a single “fear network”.

This notion is reinforced by the observed functional associations of the identified brain pattern of altered activity in panic disorder. Our meta-analytic query of the Neurosynth database has linked the functional substrates of panic disorder to aspects of memory processes, learning, and emotional arousal as well as to attentional processes, motor processes, and goal-directed behavior. Hence, symptoms of panic disorder may be linked to alterations of memory-related evaluation of potential threat stimuli, which results in inadequate emotional arousal as well as impaired attentional and goal-directed action. However, such stimuli may also evolve from signals from the organism’s internal milieu that are processed below the conscious awareness by phylogenetically older areas, including particularly the brainstem [63, 65]. More precisely, the “suffocation false alarm theory” has conceived unexpected panic attacks as a primal defensive reaction to threat within the internal milieu of the body and related them to the activation of phylogenetically older brain structures, which process basic information related to the body’s internal milieu [62, 63]. Our analyses support this theory by showing that increased activity is enriched in areas with least neurodevelopmental and evolutionary expansion and with low oxygen metabolism–including the brainstem, consistent with the suffocation false alarm hypothesis [62] and neuroimaging evidence implicating brainstem mechanisms in panic disorder [63]. Given that the brainstem (and hippocampus) holds a low metabolic rate of oxygen to oxygen delivery ratio, i.e., oxygen extraction fraction, and features a high density of central chemoreceptors [66, 67], it might be concluded in accordance with the “suffocation false alarm theory” that panic attacks result from abnormal chemosensitivity of hyperactive phylogenetically older, chemosensitive brain regions. Evidence of patients with panic disorder showing increased brainstem activation in response to experimentally induced hypercapnia compared to controls and divers suggests increased neural sensitivity to CO_2_ at the brainstem level [68].

We also find increased activity in the left dorsolateral prefrontal cortex, i.e. phylogenetically more recent transmodal cortex, to be involved in the etiopathology of panic disorder. While this may also indicate the disturbance of “higher-level” mental functions, our meta-analyses cannot distinguish psychological from physiological causes of panic disorder or even weight their respective impact on etiopathogenesis. However, the functional involvement of brainstem regions together with limbic and prefrontal areas suggests that the etiopathology of panic disorder may be based on dynamic psychophysiological interactions of higher-order cognitive systems and evolutionary older biological processes. That is, the results of our meta-analysis in combination with behavioral profiling suggest that the identified brain pattern reflects both psychological and physiological alterations linked to panic symptoms. This notion is supported by the observation that psychotherapy of panic disorder, targeting higher mental processes, can normalize hyperactivation of the left dorsolateral prefrontal cortex [69]. Given that increased pre-treatment activity of the left dorsolateral predicts better psychotherapy outcome [70], it may be inferred that overactivity may reflect ineffective regulation, which becomes more efficient following psychotherapy [69].

Especially the (putative) involvement of brainstem nuclei and the known therapeutic response to selective serotonin reuptake inhibitors fueled the notion that dysregulation of the serotonergic neurotransmitter system is central to the pathophysiology of panic disorder [71, 72]. In this context, it has been reasoned that particularly the brainstem serotonergic system is involved in panic modulation with both altered serotonergic receptors and 5-HT transporter bindings [63]. Our report of a robust association between altered brain activity and serotonin transporter as well as 5-HT_1A_ receptor distribution supports this hypothesis and complements the findings of a small-sized study providing evidence for reduced 5-HT_1A_ receptor binding in untreated panic disorder [73]. While the focus of research into brain neurotransmission in panic disorder has been on the serotonergic system, potential alterations in the dopamine system have been rather neglected. There is preliminary evidence that binding potential of the dopamine transporter reflects clinical status in panic disorder [74]. Case reports indicate that dopamine agonists may induce panic-like episodes [75, 76]. Given that blockade of D_2_ receptors facilitates, while activation inhibits fear extinction in rodents, it has been postulated that D_2_ antagonists may be useful adjuncts to therapy of human anxiety disorders [77]. However, there is only anecdotic evidence that specific blockade of the D_2_ receptor reduces panic attacks [78]. Our study complements these findings by showing that disorder-related brain activity specifically links to the D_2_ receptor and the dopamine transporter. Thus, these results also corroborate the somewhat older and long-forgotten notion of dopaminergic overactivity in panic disorder [79]. Future research should investigate whether these brain activity–chemoarchitecture associations are due to altered availability or expression of receptors/transporters. Having a look at underlying gene expression profiles may provide clues to answer this question.

It is well-known that the association of mRNA expression with protein products and receptor density may vary greatly between genes, being not associated at all or even negatively associated for some, and strongly correlated for others [80–82]. This may be due to temporal decoupling or override of the transcriptional level by other levels of regulation [81]. It is therefore noteworthy that also the results of our gene expression analyses indicate disturbance of both serotonergic (*HTR1A*, *HTR2A*, *SLC6A4*, *MAOA*) and dopaminergic (*DRD2, SLC6A3*) systems as critical factors in the pathophysiology of panic disorder. The significant associations of the panic brain pattern with the *BDKRB2*, *COMT* and *CRHR1* genes, however, point to additional involvement of sympathetic, bradykinin-induced catecholamine regulation and stress response-regulating cortisol systems. It is remarkable that we also found a link to activity of *TMEM132D*, a candidate gene that was only recently identified by a seminal report including a whole-genome association study and mouse models [83]. The function of the gene product is still unknown, but mouse models suggest that it is most prominently expressed in neurons and colocalizes with actin filaments, pointing to a major role in neuronal sprouting and connectivity in brain regions implicated in anxiety-related behavior [84, 85]. While its specificity to panic disorder in general remains to be clarified, the synopsis of several large datasets indicates that *TMEM132D* specifically contributes to genetic susceptibility for panic disorder in individuals of European ancestry [84]. Recent animal and human data suggest that life events affect methylation status of *TMEM132D*, which in turn mediates the relationship between life events and symtom severity of panic disorder [86, 87]. That is, *TMEM132D* methylation might be the substrate of gene × environment interactions in the pathophysiology of panic disorder. The most striking result of our gene association analyses, however, was that several genes significantly contributed to explain the pattern of altered brain activity in panic disorder, substantiating the notion of a polygenic condition.

Identifying robust neural alterations in panic disorder is clinically relevant for several reasons. First, these findings may inform biomarker development for diagnosis or treatment stratification. Second, the involvement of prefrontal and brainstem regions aligns with targets of neuromodulation and psychotherapy approaches that aim to enhance cognitive control and regulate autonomic responses. Future work could explore whether these neural patterns predict treatment response to interventions such as cognitive-behavioral therapy or pharmacotherapy.

Several limitations should be acknowledged. Our analyses relied on aggregated coordinates rather than raw imaging data, which limits the ability to account for individual variability. The included studies employed heterogeneous tasks and imaging protocols, which may introduce variability despite sensitivity analyses. Clinical heterogeneity across studies—including differences in medication status, comorbidities, illness duration, and agoraphobia—may contribute to variation in observed activation patterns. Because these variables were inconsistently reported in the original studies, we refrained from underpowered meta‑regressions but provide all available moderator data in Supplementary Table 1 for transparency. Furthermore, receptor and gene expression maps were derived from healthy populations, and their relevance to pathological states remains uncertain. Finally, the neurotransmission functional characterization using meta-analytic annotations is correlational and cannot establish causal relationships. Future research should validate these findings in prospective cohorts using harmonized imaging protocols and individual-level data. Longitudinal studies could clarify whether these neural alterations predict clinical trajectories or treatment outcomes. Integrating multimodal data—including neuroimaging, genetics, and behavioral measures—may help identify mechanistic pathways and guide precision interventions. Comparative analyses across anxiety disorders will also be essential to determine the specificity of these patterns to panic disorder.

Our integrative cross-modal analyses indicate that panic disorder is associated with a consistent pattern of increased activity in the prefrontal-hippocampus-brainstem axis. These findings provide a robust, large-scale map of functional alterations, suggest that panic disorder involves alterations across multiple functional domains rather than a single “fear network”, and show systematic spatial associations with serotonergic and dopaminergic systems as well as polygenic contributions, including *TMEM132D*. While these results are partly exploratory, they establish a foundation for future research aimed at validating these patterns, clarifying their clinical relevance, and investigating whether they can inform biomarker development or targeted interventions. Taken together, this work represents an important step toward integrating functional neuroanatomy, molecular architecture, and psychopathology in panic disorder.

## Supplementary information

Supplemental Material

## Data Availability

Coordinates used in the meta-analyses are available from the included studies that are referenced in the supplement. Receptor and transporter atlases are shipped with the JuSpace Toolbox (https://github.com/juryxy/JuSpace/). Neurosynth data are available at https://neurosynth.org/. Allen Human Brain Altas data are available from https://human.brain-map.org. All other brain maps are available through the neuromaps package (https://github.com/netneurolab/neuromaps/).

## References

[1] Anonymous. Homeric Hymn 19. To Pan.

[2] Paus. 10.23.7.

[3] American Psychiatric Association: *Diagnostic and statistical manual of mental disorders: DSM-5™*, *5th ed*. Arlington, VA, US: American Psychiatric Publishing, Inc.; 2013.

[4] Penninx BWJH, Pine DS, Holmes EA, Reif A. Anxiety disorders. The Lancet. 2021;397:914–27. 10.1016/S0140-6736(21)00359-7 PMC9248771 33581801

[5] de Jonge P, Roest AM, Lim CC, Florescu SE, Bromet EJ, Stein DJ, et al. Cross-national epidemiology of panic disorder and panic attacks in the world mental health surveys. Depress Anxiety. 2016;33:1155–77. 27775828 10.1002/da.22572 PMC5143159

[6] Kessler RC, Demler O, Frank RG, Olfson M, Pincus HA, Walters EE, et al. Prevalence and treatment of mental disorders, 1990 to 2003. N Engl J Med. 2005;352:2515–23. 15958807 10.1056/NEJMsa043266 PMC2847367

[7] Wittchen HU, Jacobi F, Rehm J, Gustavsson A, Svensson M, Jonsson B, et al. The size and burden of mental disorders and other disorders of the brain in Europe 2010. Eur Neuropsychopharmacol. 2011;21:655–79. 21896369 10.1016/j.euroneuro.2011.07.018

[8] Perna G, Caldirola D. Management of treatment-resistant panic disorder. Curr Treat Options Psychiatry. 2017;4:371–86. 29238651 10.1007/s40501-017-0128-7 PMC5717132

[9] Domschke K, Seuling PD, Schiele MA, Bandelow B, Batelaan NM, Bokma WA, et al. The definition of treatment resistance in anxiety disorders: a Delphi method-based consensus guideline. World Psychiatry. 2024;23:113–23. 38214637 10.1002/wps.21177 PMC10785995

[10] Konnopka A, Konig H. Economic burden of anxiety disorders: a systematic review and meta-analysis. Pharmacoeconomics. 2020;38:25–37. 31646432 10.1007/s40273-019-00849-7

[11] Mechias ML, Etkin A, Kalisch R. A meta-analysis of instructed fear studies: implications for conscious appraisal of threat. Neuroimage. 2010;49:1760–8. 19786103 10.1016/j.neuroimage.2009.09.040

[12] Fullana MA, Harrison BJ, Soriano-Mas C, Vervliet B, Cardoner N, Avila-Parcet A, et al. Neural signatures of human fear conditioning: an updated and extended meta-analysis of fMRI studies. Mol Psychiatry. 2016;21:500–8. 26122585 10.1038/mp.2015.88

[13] LeDoux JE, Pine DS. Using neuroscience to help understand fear and anxiety: a two-system framework. Am J Psychiatry. 2016;173:1083–93. 27609244 10.1176/appi.ajp.2016.16030353

[14] Lissek S, Rabin SJ, McDowell DJ, Dvir S, Bradford DE, Geraci M, et al. Impaired discriminative fear-conditioning resulting from elevated fear responding to learned safety cues among individuals with panic disorder. Behav Res Ther. 2009;47:111–8. 19027893 10.1016/j.brat.2008.10.017 PMC2758527

[15] Webler RD, Berg H, Fhong K, Tuominen L, Holt DJ, Morey RA, et al. The neurobiology of human fear generalization: meta-analysis and working neural model. Neurosci Biobehav Rev. 2021;128:421–36. 34242718 10.1016/j.neubiorev.2021.06.035

[16] Chavanne AV, Robinson OJ. The overlapping neurobiology of induced and pathological anxiety: a meta-analysis of functional neural activation. Am J Psychiatry. 2021;178:156–64. 33054384 10.1176/appi.ajp.2020.19111153 PMC7116679

[17] Hennings AC, Cooper SE, Lewis-Peacock JA, Dunsmoor JE. Pattern analysis of neuroimaging data reveals novel insights on threat learning and extinction in humans. Neurosci Biobehav Rev. 2022;142:104918. 36257347 10.1016/j.neubiorev.2022.104918 PMC11163873

[18] Kredlow MA, de Voogd LD, Phelps EA. A case for translation from the clinic to the laboratory. Perspect Psychol Sci. 2022;17:1120–49. 35245166 10.1177/17456916211039852 PMC9271534

[19] Levy I, Schiller D. Neural computations of threat. Trends Cogn Sci. 2021;25:151–71. 33384214 10.1016/j.tics.2020.11.007 PMC8084636

[20] Leonardo ED, Hen R. Anxiety as a developmental disorder. Neuropsychopharmacology. 2008;33:134–40. 17851538 10.1038/sj.npp.1301569

[21] Moraes ACN, Wijaya C, Freire R, Quagliato LA, Nardi AE, Kyriakoulis P. Neurochemical and genetic factors in panic disorder: a systematic review. Transl Psychiatry. 2024;14:294. 39025836 10.1038/s41398-024-02966-0 PMC11258274

[22] Bas-Hoogendam JM, Blackford JU, Bruhl AB, Blair KS, van der Wee NJA, Westenberg PM. Neurobiological candidate endophenotypes of social anxiety disorder. Neurosci Biobehav Rev. 2016;71:362–78. 27593443 10.1016/j.neubiorev.2016.08.040

[23] Bearden CE, Freimer NB. Endophenotypes for psychiatric disorders: ready for primetime? Trends Genet. 2006;22:306–13. 16697071 10.1016/j.tig.2006.04.004

[24] Bearden CE, Reus VI, Freimer NB. Why genetic investigation of psychiatric disorders is so difficult. Curr Opin Genet Dev. 2004;14:280–6. 15172671 10.1016/j.gde.2004.04.005

[25] Glahn DC, Knowles EE, McKay DR, Sprooten E, Raventos H, Blangero J, et al. Arguments for the sake of endophenotypes: examining common misconceptions about the use of endophenotypes in psychiatric genetics. Am J Med Genet B Neuropsychiatr Genet. 2014;165B:122–30. 24464604 10.1002/ajmg.b.32221 PMC4078653

[26] Meier SM, Deckert J. Genetics of anxiety disorders. Curr Psychiatry Rep. 2019;21:16. 30826936 10.1007/s11920-019-1002-7

[27] Müller VI, Cieslik EC, Laird AR, Fox PT, Radua J, Mataix-Cols D, et al. Ten simple rules for neuroimaging meta-analysis. Neurosci Biobehav Rev. 2018;84:151–61. 29180258 10.1016/j.neubiorev.2017.11.012 PMC5918306

[28] Page MJ, McKenzie JE, Bossuyt PM, Boutron I, Hoffmann TC, Mulrow CD, et al. The PRISMA 2020 statement: an updated guideline for reporting systematic reviews. BMJ. 2021;372:n71. 33782057 10.1136/bmj.n71 PMC8005924

[29] Laird AR, Robinson JL, McMillan KM, Tordesillas-Gutierrez D, Moran ST, Gonzales SM, et al. Comparison of the disparity between Talairach and MNI coordinates in functional neuroimaging data: validation of the Lancaster transform. Neuroimage. 2010;51:677–83. 20197097 10.1016/j.neuroimage.2010.02.048 PMC2856713

[30] Eickhoff SB, Nichols TE, Laird AR, Hoffstaedter F, Amunts K, Fox PT, et al. Behavior, sensitivity, and power of activation likelihood estimation characterized by massive empirical simulation. Neuroimage. 2016;137:70–85. 27179606 10.1016/j.neuroimage.2016.04.072 PMC4981641

[31] Eickhoff SB, Bzdok D, Laird AR, Kurth F, Fox PT. Activation likelihood estimation meta-analysis revisited. Neuroimage. 2012;59:2349–61. 21963913 10.1016/j.neuroimage.2011.09.017 PMC3254820

[32] Turkeltaub PE, Eickhoff SB, Laird AR, Fox M, Wiener M, Fox P. Minimizing within-experiment and within-group effects in activation likelihood estimation meta-analyses. Hum Brain Mapp. 2012;33:1–13. 21305667 10.1002/hbm.21186 PMC4791073

[33] Eickhoff SB, Laird AR, Grefkes C, Wang LE, Zilles K, Fox PT. Coordinate-based activation likelihood estimation meta-analysis of neuroimaging data: a random-effects approach based on empirical estimates of spatial uncertainty. Hum Brain Mapp. 2009;30:2907–26. 19172646 10.1002/hbm.20718 PMC2872071

[34] Eickhoff SB, Heim S, Zilles K, Amunts K. Testing anatomically specified hypotheses in functional imaging using cytoarchitectonic maps. Neuroimage. 2006;32:570–82. 16781166 10.1016/j.neuroimage.2006.04.204

[35] Eickhoff SB, Paus T, Caspers S, Grosbras MH, Evans AC, Zilles K, et al. Assignment of functional activations to probabilistic cytoarchitectonic areas revisited. Neuroimage. 2007;36:511–21. 17499520 10.1016/j.neuroimage.2007.03.060

[36] Eickhoff SB, Stephan KE, Mohlberg H, Grefkes C, Fink GR, Amunts K, et al. A new SPM toolbox for combining probabilistic cytoarchitectonic maps and functional imaging data. Neuroimage. 2005;25:1325–35. 15850749 10.1016/j.neuroimage.2004.12.034

[37] Lancaster JL, Woldorff MG, Parsons LM, Liotti M, Freitas CS, Rainey L, et al. Automated Talairach atlas labels for functional brain mapping. Hum Brain Mapp. 2000;10:120–31. 10912591 10.1002/1097-0193(200007)10:3<120::AID-HBM30>3.0.CO;2-8 PMC6871915

[38] Collins DL, Holmes CJ, Peters TM, Evans AC. Automatic 3-D model-based neuroanatomical segmentation. Human Brain Mapping. 1995;3:190–208.

[39] Dukart J, Holiga S, Rullmann M, Lanzenberger R, Hawkins PCT, Mehta MA, et al. JuSpace: a tool for spatial correlation analyses of magnetic resonance imaging data with nuclear imaging derived neurotransmitter maps. Hum Brain Mapp. 2021;42:555–66. 33079453 10.1002/hbm.25244 PMC7814756

[40] Gryglewski G, Seiger R, James GM, Godbersen GM, Komorowski A, Unterholzner J, et al. Spatial analysis and high resolution mapping of the human whole-brain transcriptome for integrative analysis in neuroimaging. Neuroimage. 2018;176:259–67. 29723639 10.1016/j.neuroimage.2018.04.068

[41] Gottschalk MG, Domschke K. Genetics of anxiety disorders. In: Geddes JR, Andreasen NC, Goodwin GM, Geddes JR, Andreasen NC, Goodwin GM (eds). *New Oxford Textbook of Psychiatry*. Oxford: Oxford University Press 2020, p 928–937.

[42] Howe AS, Buttenschon HN, Bani-Fatemi A, Maron E, Otowa T, Erhardt A, et al. Candidate genes in panic disorder: meta-analyses of 23 common variants in major anxiogenic pathways. Mol Psychiatry. 2016;21:665–79. 26390831 10.1038/mp.2015.138

[43] Morimoto Y, Ono S, Kurotaki N, Imamura A, Ozawa H. Genetic and epigenetic analyses of panic disorder in the post-GWAS era. J Neural Transm (Vienna). 2020;127:1517–26. 32388794 10.1007/s00702-020-02205-y PMC7578165

[44] Azen R, Budescu DV. The dominance analysis approach for comparing predictors in multiple regression. Psychol Methods. 2003;8:129–48. 12924811 10.1037/1082-989x.8.2.129

[45] Markello RD, Hansen JY, Liu ZQ, Bazinet V, Shafiei G, Suarez LE, et al. neuromaps: structural and functional interpretation of brain maps. Nat Methods. 2022;19:1472–9. 36203018 10.1038/s41592-022-01625-w PMC9636018

[46] Hill J, Inder T, Neil J, Dierker D, Harwell J, Van Essen D. Similar patterns of cortical expansion during human development and evolution. Proc Natl Acad Sci USA. 2010;107:13135–40. 20624964 10.1073/pnas.1001229107 PMC2919958

[47] Wei Y, de Lange SC, Scholtens LH, Watanabe K, Ardesch DJ, Jansen PR, et al. Genetic mapping and evolutionary analysis of human-expanded cognitive networks. Nat Commun. 2019;10:4839. 31649260 10.1038/s41467-019-12764-8 PMC6813316

[48] Xu T, Nenning KH, Schwartz E, Hong SJ, Vogelstein JT, Goulas A, et al. Cross-species functional alignment reveals evolutionary hierarchy within the connectome. Neuroimage. 2020;223:117346. 32916286 10.1016/j.neuroimage.2020.117346 PMC7871099

[49] Vaishnavi SN, Vlassenko AG, Rundle MM, Snyder AZ, Mintun MA, Raichle ME. Regional aerobic glycolysis in the human brain. Proc Natl Acad Sci USA. 2010;107:17757–62. 20837536 10.1073/pnas.1010459107 PMC2955101

[50] Markello RD, Misic B. Comparing spatial null models for brain maps. Neuroimage. 2021;236:118052. 33857618 10.1016/j.neuroimage.2021.118052

[51] Yarkoni T, Poldrack RA, Nichols TE, Van Essen DC, Wager TD. Large-scale automated synthesis of human functional neuroimaging data. Nat Methods. 2011;8:665–70. 21706013 10.1038/nmeth.1635 PMC3146590

[52] Hansen JY, Markello RD, Vogel JW, Seidlitz J, Bzdok D, Misic B. Mapping gene transcription and neurocognition across human neocortex. Nat Hum Behav. 2021;5:1240–50. 33767429 10.1038/s41562-021-01082-z

[53] Poldrack RA, Kittur A, Kalar D, Miller E, Seppa C, Gil Y, et al. The cognitive atlas: toward a knowledge foundation for cognitive neuroscience. Front Neuroinform. 2011;5:17. 21922006 10.3389/fninf.2011.00017 PMC3167196

[54] Etkin A, Wager TD. Functional neuroimaging of anxiety: a meta-analysis of emotional processing in PTSD, social anxiety disorder, and specific phobia. Am J Psychiatry. 2007;164:1476–88. 17898336 10.1176/appi.ajp.2007.07030504 PMC3318959

[55] Janiri D, Moser DA, Doucet GE, Luber MJ, Rasgon A, Lee WH, et al. Shared neural phenotypes for mood and anxiety disorders: a meta-analysis of 226 task-related functional imaging studies. JAMA Psychiatry. 2020;77:172–9. 31664439 10.1001/jamapsychiatry.2019.3351 PMC6822098

[56] Crossley NA, Mechelli A, Ginestet C, Rubinov M, Bullmore ET, McGuire P. Altered hub functioning and compensatory activations in the connectome: a meta-analysis of functional neuroimaging studies in schizophrenia. Schizophr Bull. 2016;42:434–42. 26472684 10.1093/schbul/sbv146 PMC4753609

[57] Muller VI, Cieslik EC, Serbanescu I, Laird AR, Fox PT, Eickhoff SB. Altered brain activity in unipolar depression revisited: meta-analyses of neuroimaging studies. JAMA Psychiatry. 2017;74:47–55. 27829086 10.1001/jamapsychiatry.2016.2783 PMC5293141

[58] Poeppl TB, Donges MR, Mokros A, Rupprecht R, Fox PT, Laird AR, et al. A view behind the mask of sanity: meta-analysis of aberrant brain activity in psychopaths. Mol Psychiatry. 2019;24:463–70. 30038232 10.1038/s41380-018-0122-5 PMC6344321

[59] Schnellbächer GJ, Dukart J, Hansen JY, Markello RD, Mokros A, Pietsch V, et al. Aberrant brain activity in pedophilia links to receptor distribution, gene expression, and behavior. Nat Mental Health. 2023;1:615–22.

[60] Hansen JY, Shafiei G, Markello RD, Smart K, Cox SML, Norgaard M, et al. Mapping neurotransmitter systems to the structural and functional organization of the human neocortex. Nat Neurosci. 2022;25:1569–81. 36303070 10.1038/s41593-022-01186-3 PMC9630096

[61] Hawrylycz MJ, Lein ES, Guillozet-Bongaarts AL, Shen EH, Ng L, Miller JA, et al. An anatomically comprehensive atlas of the adult human brain transcriptome. Nature. 2012;489:391–9. 22996553 10.1038/nature11405 PMC4243026

[62] Klein DF. False suffocation alarms, spontaneous panics, and related conditions. an integrative hypothesis. Arch Gen Psychiatry. 1993;50:306–17. 8466392 10.1001/archpsyc.1993.01820160076009

[63] Perna G, Guerriero G, Brambilla P, Caldirola D. Panic and the brainstem: clues from neuroimaging studies. CNS Neurol Disord Drug Targets. 2014;13:1049–56. 24923341 10.2174/1871527313666140612112923

[64] Fullana MA, Albajes-Eizagirre A, Soriano-Mas C, Vervliet B, Cardoner N, Benet O, et al. Fear extinction in the human brain: a meta-analysis of fMRI studies in healthy participants. Neurosci Biobehav Rev. 2018;88:16–25. 29530516 10.1016/j.neubiorev.2018.03.002

[65] Damasio A. *The feeling of what happens: body and emotion in the making of consciousness*. New York: Harcourt Brace and Co; 1999.

[66] Henriksen OM, Gjedde A, Vang K, Law I, Aanerud J, Rostrup E. Regional and interindividual relationships between cerebral perfusion and oxygen metabolism. Journal of Applied Physiology. 2021;130:1836–47. 33830816 10.1152/japplphysiol.00939.2020

[67] Nattie E, Li A. Central chemoreceptors: locations and functions. Compr Physiol. 2012;2:221–54. 23728974 10.1002/cphy.c100083 PMC4802370

[68] Goossens L, Leibold N, Peeters R, Esquivel G, Knuts I, Backes W, et al. Brainstem response to hypercapnia: a symptom provocation study into the pathophysiology of panic disorder. Journal of Psychopharmacology. 2014;28:449–56. 24646808 10.1177/0269881114527363

[69] Reinecke A, Thilo KV, Croft A, Harmer CJ. Early effects of exposure-based cognitive behaviour therapy on the neural correlates of anxiety. Transl Psychiatry. 2018;8:225. 30341276 10.1038/s41398-018-0277-5 PMC6195621

[70] Reinecke A, Thilo K, Filippini N, Croft A, Harmer CJ. Predicting rapid response to cognitive-behavioural treatment for panic disorder: the role of hippocampus, insula, and dorsolateral prefrontal cortex. Behav Res Ther. 2014;62:120–8. 25156399 10.1016/j.brat.2014.07.017

[71] Gorman JM, Kent JM, Sullivan GM, Coplan JD. Neuroanatomical hypothesis of panic disorder, revised. Am J Psychiatry. 2000;157:493–505. 10739407 10.1176/appi.ajp.157.4.493

[72] Grove G, Coplan JD, Hollander E. The neuroanatomy of 5-HT dysregulation and panic disorder. J Neuropsychiatry Clin Neurosci. 1997;9:198–207. 9144099 10.1176/jnp.9.2.198

[73] Nash JR, Sargent PA, Rabiner EA, Hood SD, Argyropoulos SV, Potokar JP, et al. Serotonin 5-HT1A receptor binding in people with panic disorder: positron emission tomography study. Br J Psychiatry. 2008;193:229–34. 18757983 10.1192/bjp.bp.107.041186

[74] Maron E, Nutt DJ, Kuikka J, Tiihonen J. Dopamine transporter binding in females with panic disorder may vary with clinical status. J Psychiatr Res. 2010;44:56–59. 19467545 10.1016/j.jpsychires.2009.04.014

[75] Alonso-Navarro H, Jimenez-Jimenez FJ. Panic attack like episodes possibly associated with pramipexole therapy in Parkinson’s disease. Eur J Neurol. 2007;14:e1. 17437590 10.1111/j.1468-1331.2007.01715.x

[76] Alonso-Navarro H, Jimenez-Jimenez FJ, Pilo-de-la-Fuente B, Plaza-Nieto JF. Panic attack-like episodes possibly associated with ropinirole. Clin Neuropharmacol. 2009;32:237–8. 19644237 10.1097/01.wnf.0000265980.03198.55

[77] Ponnusamy R, Nissim HA, Barad M. Systemic blockade of D2-like dopamine receptors facilitates extinction of conditioned fear in mice. Learn Mem. 2005;12:399–406. 16077018 10.1101/lm.96605 PMC1183258

[78] Takahashi H, Sugita T, Yoshida K, Higuchi H, Shimizu T. Effect of quetiapine in the treatment of panic attacks in patients with schizophrenia: 3 case reports. J Neuropsychiatry Clin Neurosci. 2004;16:113–5. 14990767 10.1176/jnp.16.1.113

[79] Pitchot W, Ansseau M, Gonzalez Moreno A, Hansenne M, von Frenckell R. Dopaminergic function in panic disorder: comparison with major and minor depression. Biol Psychiatry. 1992;32:1004–11. 1467380 10.1016/0006-3223(92)90061-4

[80] Hansen JY, Markello RD, Tuominen L, Norgaard M, Kuzmin E, Palomero-Gallagher N, et al. Correspondence between gene expression and neurotransmitter receptor and transporter density in the human brain. Neuroimage. 2022;264:119671. 36209794 10.1016/j.neuroimage.2022.119671

[81] Koussounadis A, Langdon SP, Um IH, Harrison DJ, Smith VA. Relationship between differentially expressed mRNA and mRNA-protein correlations in a xenograft model system. Sci Rep. 2015;5:10775. 26053859 10.1038/srep10775 PMC4459080

[82] Moritz CP, Muhlhaus T, Tenzer S, Schulenborg T, Friauf E. Poor transcript-protein correlation in the brain: negatively correlating gene products reveal neuronal polarity as a potential cause. J Neurochem. 2019;149:582–604. 30664243 10.1111/jnc.14664

[83] Erhardt A, Czibere L, Roeske D, Lucae S, Unschuld PG, Ripke S, et al. TMEM132D, a new candidate for anxiety phenotypes: evidence from human and mouse studies. Mol Psychiatry. 2011;16:647–63. 20368705 10.1038/mp.2010.41

[84] Erhardt A, Akula N, Schumacher J, Czamara D, Karbalai N, Muller-Myhsok B, et al. Replication and meta-analysis of TMEM132D gene variants in panic disorder. Transl Psychiatry. 2012;2:e156. 22948381 10.1038/tp.2012.85 PMC3565207

[85] Walser SM, Dedic N, Touma C, Floss T, Wurst W, Holsboer F, et al. TMEM132D – a putative cell adhesion molecule involved in panic disorder. Pharmacopsychiatry. 2011;21:A117.

[86] Naik RR, Sotnikov SV, Diepold RP, Iurato S, Markt PO, Bultmann A, et al. Polymorphism in Tmem132d regulates expression and anxiety-related behavior through binding of RNA polymerase II complex. Transl Psychiatry. 2018;8:1. 29317594 10.1038/s41398-017-0025-2 PMC5802467

[87] Yu Q, Wang C, Xu H, Wu Y, Ding H, Liu N, et al. The mediating role of transmembrane protein 132D methylation in predicting the occurrence of panic disorder in physical abuse. Front Psychiatry. 2022;13:972522. 36032246 10.3389/fpsyt.2022.972522 PMC9403743

