# Supplementary material for: Beyond fear circuits: multiscale neurobiological architecture of panic disorder
Source: Mol Psychiatry. 2026 May 27;31(10):5800–10. doi: 10.1038/s41380-026-03661-w (PMC13569418; doi:10.1038/s41380-026-03661-w)
Supplement: Supplementary file 1 [file 41380_2026_3661_MOESM1_ESM.docx]

**Supplementary Material**

**Supplementary Methods**

We employed the revised activation likelihood estimation (ALE) algorithm (Eickhoff et al., 2012): Foci are organized by the experiment (and per subject group) that reported them. Gaussian widths are calculated based on empirical quantification of the uncertainty inherent in spatial normalization, and the relationship between sample size and inter-subject localization uncertainty. An individual map of activation likelihood, called a Modeled Activation map (MA map), is then calculated for each experiment by taking the maximum probability associated with any one focus reported by the experiment. This always corresponds to the probability of the focus with the shortest Euclidean distance to the voxel in question. In this way, MA maps reflect the spatial distribution of activation likelihoods associated with reported foci, without allowing multiple foci from one experiment to jointly influence the individual MA value of a single voxel. The ALE map is then calculated as the voxel-wise union of the probabilities in all the MA maps from a dataset. Null distributions account for the increased likelihood of identifying activation foci in gray matter.

We relied on the revised activation likelihood estimation (ALE) approach for computing the null-distribution (Eickhoff et al., 2012). The key idea behind this revised analytical solution was to abandon the previously employed permutation procedure in favor of a non-linear histogram integration, which could be described as a weighted convolution (cf., [Hope, 1968](https://doi.org/10.1111/j.2517-6161.1968.tb00759.x)). To this end, the computational unit of the revised algorithm is not distinct voxels but distinct modeled activation (MA)-values. That is, rather than considering each voxel individually, all voxels showing the same MA-value in a particular experiment are joined into and represented as a single histogram-bin. The entire histogram thus holds the occurrences of all possible MA-values (including those that are zero (voxels not in the vicinity of any reported focus)) in form of bins, summarizing the MA-map without its spatial information. These histograms are then successively merged throughout the different experiments considered in the meta-analysis to derive the null-distribution of ALE-values under spatial independence. This approach takes advantage of the fact that the number of unique MA-values in each map is considerably smaller than the number of voxels, i.e., that many voxels show the same MA-value. The proposed algorithm thus represents a special case of a permutation test, where each the pool of values that may be drawn from each individual experiment may be represented parsimoniously by the probabilities for the (limited number of) different values. This allows to analytically compute rather than to empirically collect the probabilities of possible outcomes in the permutation test.

In order to compute the null-distribution of ALE values under spatial independence, each MA-map is first converted into a histogram of observed values. The bin width of these histograms is set to 0.00001 (unit being MA-values, i.e., activation probabilities). Each histogram is then normalized to a sum of one, rendering the histogram-values probabilities of observing the MA-value corresponding to this particular bin in the respective MA-map. The histogram of the null distribution is initialized to correspond to a flat prior with all probabilities being zero. In order to derive final histogram of ALE-values under the null-hypothesis, the histograms corresponding to the MA-maps of the individual experiments are then successively combined. That is, initially, an ALE-histogram is computed by integrating the histograms of the first two experiments. The resulting ALE-histogram is then merged with the normalised histogram representing the MA-values of experiment three. Again, the output histogram is initialized to contain only zeros and the filled as described below. The histogram resulting from the successive integration of the histograms representing the MA-maps of the first three experiments is combined in the same fashion with the one of experiment four and so on. As this integration fulfils associativity like any multiplication, the order of which the MA maps are combined is irrelevant to this calculation. Once all experiments are considered, the final ALE-histogram representing the null-hypothesis for statistical inference is derived. In this context, it is important to note MA- and ALE-values are conceptually identical, as both represent the probability of an activation being present at a given voxel. This equivalence is highlighted by the fact that the probability information of the individual foci reported in a particular experiment is combined into an MA-map in exactly the same fashion (computing the voxel-wise union of probabilities), as MA-maps from different experiments are combined into an ALE-map. The difference in nomenclature thus purely reflects the difference between data pertaining to a single experiment (MA-values) and data computed by the combination of information from different individual experiments (ALE-values).

This analytical solution was implemented as follows: As noted above, all bins of the output-histogram were initialized to a have a probability of zero. The integration algorithm used for combining two MA- or ALE-histograms (here denoted *a* and *b*) into a joint (output) histogram *c* involves cycling through all non-zero bins of both histograms. Each pair of bins is then combined according to the following algorithm. Let *b_j_* be the current bin, i.e., MA- or ALE-value, of the first histogram and *p_j_^a^* the corresponding probability. Likewise, *b_k_* denotes the current bin, i.e., MA- or ALE-value, of the second histogram and *p_k_^b^* the corresponding probability. The ALE-value *l* that would be observed in the resulting ALE-map *c* when voxels drawn from these two bins, *b_j_* and *b_k_*, are combined is given by the union of these, i.e., *l* = 1−[(1−*b_j_*) ∗ (1−*b_k_*)], whilst its corresponding bin in the output-histogram is *b_l_*. The probability *p_l_* of these two bins being conjointly present in a random association, e.g., when drawing voxels at random from both maps, is given by *p_j_^a^* ∗ *p_k_^b^*. This probability *p_l_* can be conceptualized as the probability of drawing by chance a voxel from MA- or ALE-map *a* that has a value of *b_j_*, and simultaneously drawing a voxel from MA- or ALE-map *b* that has a value of *b_k_*. As a final step, the probability *p_l_^c^* for the bin *b_l_* in the output-histogram is incremented by the observed probability, i.e., *p_l_^c^* = *p_l_^c^* + *p_l_*. This process is continued until all non-zero bins of both input-histograms (representing the result of the previous integration and the next MA-map, respectively) have been combined with each other. The resulting output-histogram now represents the probabilistic distribution of ALE-values resulting from a random combination of the ALE- or MA-maps represented by the two input-histograms, initially derived from two experiments' sets of activation foci.

We then test for statistical significance using threshold-free cluster enhancement (TFCE) correction at *p*<0.05, which is based on permutation testing to determine a threshold value as follows: First, an empty version of the meta-analytic dataset (i.e., featuring a given number of experiments, each with a given number of subjects and foci) is filled with random foci sampled from the gray- matter mask. Second, ALE is run on it. Third, the highest TFCE value is served. These steps are repeated 10,000 times. This gives a distribution of maximum values under the assumption of spatial independence, from which we select the value representing the 95th percentile as a significance threshold for the results of the original analysis. This way a family-wise error rate of 5% is established.

**Supplementary Figures**

**Identification of studies via databases and registers**

Records removed *before screening*:

Duplicate records removed (n = 0)

Records identified from:

Databases (n = 842)

Other sources (n = 0)

**Identification**

Records excluded

(n = 713)

Records screened

(n = 842)

Reports sought for retrieval

(n = 129)

Reports not retrieved

(n = 0)

**Screening**

Reports excluded:

No patients (n = 11)

No disorder-isolating analysis (n = 17)

No coordinates (n = 13)

Region-of-interest analysis (n = 41)

No functional imaging (n = 13)

Reports assessed for eligibility

(n = 129)

Studies included in meta-analytic review

(n = 34)

**Included**

**Supplementary Figure 1**. PRISMA 2020 flow diagram.

**Supplementary Figure 2**. Summary of the 620 activation foci reported in all 106 experiments included in the meta-analyses.

**Supplementary Figure 3**. Activation likelihood estimates (ALE), reflecting, for each voxel, the union of the modeled activation (MA) maps across all experiments reporting aberrant brain activity associated with panic disorder.

**Supplementary Figure 4**. Activation likelihood estimates (ALE), reflecting, for each voxel, the union of the modeled activation (MA) maps across experiments involving emotional stimuli.

**Supplementary Figure 5**. Activation likelihood estimates (ALE), reflecting, for each voxel, the union of the modeled activation (MA) maps across experiments not involving emotional stimuli.

**Supplementary Figure 6**. Activation likelihood estimates (ALE), reflecting, for each voxel, the union of the modeled activation (MA) maps across experiments involving learning.

**Supplementary Figure 7**. Activation likelihood estimates (ALE), reflecting, for each voxel, the union of the modeled activation (MA) maps across experiments not involving learning.

**Supplementary Tables**

**Supplementary Table 1**

| **First author** | **Year** | **% female** | **Illness duration [m]** | **% medicated** | **% w/ agoraphobia** | **Specific other comorbidities** | **% w/ comorbidities** |
| --- | --- | --- | --- | --- | --- | --- | --- |
| Ball | 2013 | 83 | n.r. | 0 | n.r. | MDD, OCD, social phobia | 28 |
| Boshuisen | 2002 | 71 | n.r. | 0 | n.r. | No psychiatric comorbidity | 0 |
| Chechko | 2009 | 56 | 137 | 100 | 78 | Major/specific psychiatric comorbidities excluded | n.r. |
| Dresler | 2012 | 55 | n.r. | 60 | 50 | Dysthymia, MDD, OCD, somatoform disorder, specific or social phobia | 65 |
| Engel | 2016 | 43 | n.r. | 52 | 100 | Major/specific psychiatric comorbidities excluded | n.r. |
| Feldker | 2016 | 77 | n.r. | 0 | 54 | BN, GAD, MDD, OCD, somatoform disorder, specific or social phobia | 50 |
| Feldker | 2018 | 77 | n.r. | 0 | 50 | BN, GAD, MDD, OCD, somatoform disorder, specific or social phobia | 46 |
| Fonzo | 2015 | 67 | n.r. | 0 | n.r. | Major/specific psychiatric comorbidities excluded | n.r. |
| Gechter | 2019 | 65 | n.r. | 0 | 100 | Major/specific psychiatric comorbidities excluded | n.r. |
| Gorka | 2014 | 77 | n.r. | n.r. | n.r. | MDD, OCD, PTSD, specific or social phobia | 100 |
| Held-Poschardt | 2018 | 70 | n.r. | 0 | n.r. | Major/specific psychiatric comorbidities excluded | n.r. |
| Heuvel | 2011 | 50 | n.r. | 0 | n.r. | MDD; Major/specific psychiatric comorbidities excluded | 25 |
| Jin | 2020 | 67 | n.r. | 0 | n.r. | No psychiatric comorbidity | 0 |
| Kaldewaij | 2019 | 78 | n.r. | 0 | 56 | Specific phobia | 17 |
| Kircher | 2013 | 69 | n.r. | 0 | 100 | Major psychiatric comorbidities excluded | 74 |
| Korgaonkar | 2021 | 73 | n.r. | 50 | 64 | GAD, MDD, OCD, PTSD, social phobia | >64 |
| Liebscher | 2016 | 66 | n.r. | 0 | 100 | Major/specific psychiatric comorbidities excluded | n.r. |
| Lueken | 2014 | 68 | n.r. | 0 | 100 | Major/specific psychiatric comorbidities excluded | n.r. |
| Maddock | 2003 | 67 | n.r. | 0 | 83 | No psychiatric comorbidity | 0 |
| Magnotta | 2014 | 69 | 174 | 77 | n.r. | Not reported | n.r. |
| McIntosh | 2020 | 62 | n.r. | n.r. | 64 | MDD; Major psychiatric comorbidities excluded | n.r. |
| Meyer | 2000 | 100 | n.r. | 0 | 67 | Not reported | n.r. |
| Petrowski | 2014 | 87 | n.r. | 0 | 100 | No psychiatric comorbidity | 0 |
| Pfleiderer | 2010 | 50 | n.r. | 50 | 35 | MDD, social phobia | 45 |
| Reinecke | 2015 | 78 | n.r. | 0 | 56 | Specific phobia; Major psychiatric comorbidities excluded | 17 |
| Ridderbusch | 2019 | 62 | n.r. | 0 | 100 | Major/specific psychiatric comorbidities excluded | n.r. |
| Ridderbusch | 2020 | 75 | n.r. | 0 | 100 | Major/specific psychiatric comorbidities excluded | n.r. |
| Schwarzmeier | 2019 | 70 | n.r. | 0 | n.r. | Major/specific psychiatric comorbidities excluded | n.r. |
| Straube | 2014 | 75 | n.r. | 0 | 100 | Major/specific psychiatric comorbidities excluded | n.r. |
| Wade-B. | 2020 | 70 | n.r. | n.r. | 55 | Dysthymia, GAD, MDD, OCD, specific or social phobia | n.r. |
| Wang | 2021 | 50 | n.r. | 0 | n.r. | GAD, MDD, social phobia | 35 |
| Wintermann | 2013 | 46 | n.r. | 0 | 100 | No psychiatric comorbidity | 0 |
| Wittmann | 2018 | 67 | n.r. | 0 | 100 | Major/specific psychiatric comorbidities excluded | n.r. |
| Yang | 2020 | 57 | n.r. | 0 | n.r. | Major/specific psychiatric comorbidities excluded | n.r. |

BN, Bulimia nervosa. GAD, Generalized anxiety disorder. n.r., not reported. MDD, Major depressive disorder. OCD, Obsessive compulsive disorder. PTSD, Post-traumatic stress disorder.

**Supplementary Table 2**

Studies reporting functional neuroimaging experiments on alterations in brain activity associated with panic disorder

| **First author** | **Year** | **Subjects** | | **Imaging** | **Task** | **Subject-Level Analysis** | **Group-Level Analysis** | **Foci** | **Direction** |
| --- | --- | --- | --- | --- | --- | --- | --- | --- | --- |
|  |  | PD | HC |  |  |  |  |  |  |
| Ball | 2013 | 18 | 22 | fMRI | Emotion regulation ^E^ | Maintain vs. Baseline | Controls > Panic disorder | 21 | - |
|  |  | 18 | 22 | fMRI | Emotion regulation ^E^ | Reappraise vs. Baseline | Controls > Panic disorder | 7 | - |
| Boshuisen | 2002 | 17 | 21 | PET | Induced anticipatory anxiety ^E^ | Anticipatory anxiety | Controls > Panic disorder | 4 | - |
|  |  | 17 | 21 | PET | Induced anticipatory anxiety ^E^ | Anticipatory anxiety | Panic disorder > Controls | 4 | + |
|  |  | 17 | 21 | PET | Induced anticipatory anxiety ^E^ | Anticipatory anxiety | Group × Time | 1 |  |
| Chechko | 2009 | 18 | 18 | fMRI | Stroop-Emotional ^E^ | Stroop_con | Panic disorder > Controls | 1 | + |
|  |  | 18 | 18 | fMRI | Stroop-Emotional ^E^ | Stroop_con | Controls > Panic disorder | 1 | - |
|  |  | 18 | 18 | fMRI | Stroop-Emotional ^E^ | Stroop_inc | Panic disorder > Controls | 1 | + |
|  |  | 18 | 18 | fMRI | Stroop-Emotional ^E^ | Stroop_inc | Controls > Panic disorder | 1 | - |
|  |  | 18 | 18 | fMRI | Stroop-Emotional ^E^ | Stroop_inc > Stroop_con | Panic disorder > Controls | 2 | + |
|  |  | 18 | 18 | fMRI | Stroop-Emotional ^E^ | Stroop_inc > Stroop_con | Controls > Panic disorder | 1 | - |
|  |  | 18 | 18 | fMRI | Stroop-Emotional ^E^ | cC > iC | Panic disorder > Controls | 2 | + |
| Dresler | 2012 | 20 | 23 | fMRI | Stroop-Emotional ^E^ | Panic-related > Neutral | Panic disorder > Controls | 1 | + |
| Engel | 2016 | 21 | 21 | fMRI | Emotion induction ^E^ | Aversive > Neutral | Controls > Panic disorder | 3 | - |
|  |  | 21 | 21 | fMRI | Emotion induction ^E^ | Panic-specific > Neutral | Panic disorder > Controls | 5 | + |
|  |  | 21 | 21 | fMRI | Emotion induction ^E^ | Panic-specific > Aversive | Panic disorder > Controls | 7 | + |
| Feldker | 2016 | 26 | 26 | fMRI | Emotion induction ^E^ | Disorder-related > Neutral | Panic disorder > Controls | 31 | + |
|  |  | 26 | 26 | fMRI | Emotion induction ^E^ | Disorder-related > Neutral | Controls > Panic disorder | 1 | - |
| Feldker | 2018 | 26 | 26 | fMRI | Target distractor task ^E^ | Panic-related > Neutral | Panic disorder > Controls | 11 | + |
| Fonzo | 2015 | 15 | 15 | fMRI | Facial emotion matching ^E^ | Fearful/Angry/Happy vs. shapes | Panic disorder > Controls | 5 | + |
|  |  | 15 | 15 | fMRI | Facial emotion matching ^E^ | Fearful/Angry/Happy vs. shapes | Controls > Panic disorder | 1 | - |
|  |  | 15 | 15 | fMRI | Facial emotion matching ^E^ | Fearful vs. Happy | Panic disorder > Controls | 1 | + |
|  |  | 15 | 15 | fMRI | Facial emotion matching ^E^ | Angry vs. Happy | Panic disorder > Controls | 2 | + |
| Gechter | 2019 | 31 | 19 | fMRI | Emotion induction ^E^ | Panic > Neutral cue | Diagnosis × Genotype | 3 |  |
| Gorka | 2014 | 13 | 18 | fMRI | Reward anticipation task | Reward > No incentive | Controls > Panic disorder | 1 | - |
| Held-Poschardt | 2018 | 10 | 10 | fMRI | Monetary incentive delay task | Maximal Loss vs. Gain anticipation | Panic disorder > Controls | 1 | + |
| Heuvel | 2011 | 14 | 22 | fMRI | Tower of London | Planning vs. Baseline | Controls > Panic disorder | 3 | - |
|  |  | 14 | 22 | fMRI | Tower of London | Task load | Controls > Panic disorder | 2 | - |
| Jin | 2020 | 18 | 21 | fMRI | Interoceptive perception | Heartbeat > Rest | Panic disorder > Controls | 4 | + |
|  |  | 18 | 21 | fMRI | Exteroceptive perception | Pure-tone > Rest | Panic disorder > Controls | 12 | + |
| Kaldewaij | 2019 | 18 | 17 | fMRI | Emotional face perception ^E^ | Fearful/Happy/Neutral vs. Baseline | Panic disorder > Controls | 1 | + |
|  |  | 18 | 17 | fMRI | Emotional face perception ^E^ | Happy vs. Neutral | Panic disorder > Controls | 1 | + |
|  |  | 18 | 17 | fMRI | Emotional face perception ^E^ | Fearful vs. Happy | Controls > Panic disorder | 3 | - |
| Kircher | 2013 | 42 | 42 | fMRI | Fear conditioning ^E, L^ | CS+ unpaired > CS- | Panic disorder > Controls | 1 | + |
|  |  | 42 | 42 | fMRI | Fear conditioning ^E, L^ | CS+ unpaired > CS- | Group × Time | 1 |  |
| Korgaonkar | 2021 | 22 | 33 | fMRI | Emotional face processing ^E^ | Supraliminal emotion processing (FWE) | Main effect of diagnosis | 2 |  |
|  |  | 22 | 33 | fMRI | Emotional face processing ^E^ | Subliminal emotion processing (unc.) | Main effect of diagnosis | 18 |  |
|  |  | 22 | 33 | fMRI | Emotional face processing ^E^ | Subliminal emotion processing (unc.) | Emotion × Group | 8 |  |
| Liebscher | 2016 | 94 | 29 | fMRI | Emotion induction ^E^ | Panic > Neutral | Panic disorder > Controls | 23 | + |
|  |  | 94 | 29 | fMRI | Emotion induction ^E^ | Panic > Neutral | Controls > Panic disorder | 12 | - |
| Lueken | 2014 | 60 | 60 | fMRI | Fear conditioning (differential) ^E, L^ | Acquisition: CS+ unpaired > CS- | Panic disorder > Controls | 5 | + |
|  |  | 60 | 60 | fMRI | Fear conditioning (simple) ^E, L^ | CS+ unpaired (acquisition) > CS+ (familiarization) | Panic disorder > Controls | 4 | + |
|  |  | 60 | 60 | fMRI | Fear conditioning (sfty learning) ^E, L^ | CS- (acquisition) > CS- (familiarization) | Panic disorder > Controls | 4 | + |
|  |  | 60 | 60 | fMRI | Fear conditioning ^E, L^ | CS+ unpair. + CS- (acqu.) > CS+ + CS- (famil.) (conj.) | Panic disorder > Controls | 2 | + |
| Maddock | 2003 | 6 | 8 | fMRI | Emotion induction ^E^ | Threat-related > Neutral | Panic disorder > Controls | 2 | + |
|  |  | 6 | 8 | fMRI | Emotion induction ^E^ | Threat-related > Neutral | Controls > Panic disorder | 8 | - |
| Magnotta | 2014 | 13 | 14 | fMRI | Flashing checkerboard | Flashing checkerboard (T1p) | Panic disorder > Controls | 1 | + |
|  |  | 13 | 14 | fMRI | Flashing checkerboard | Flashing checkerboard (T1p) | Controls > Panic disorder | 1 | - |
|  |  | 13 |  | fMRI | Flashing checkerboard | Flashing checkerboard (T1p) | Positive correlation w/ BAI | 2 | + |
|  |  | 13 |  | fMRI | Flashing checkerboard | Flashing checkerboard (T1p) | Negative correlation w/ BAI | 1 | - |
| McIntosh | 2020 | 21 | 21 | fMRI | Breath holding | Breath-hold > Rest | Panic disorder > Controls | 12 | + |
| Meyer | 2000 | 9 | 18 | PET | Visual continuous performance task | Before D-fenfluramine administration | Controls > Panic disorder | 2 | - |
|  |  | 9 | 18 | PET | Visual continuous performance task | After D-fenfluramine administration | Panic disorder > Controls | 2 | + |
| Petrowski | 2014 | 15 | 15 | fMRI | Decision task | Faces/Places | Main effect of diagnosis | 12 |  |
|  |  | 15 | 15 | fMRI | Decision task | Faces vs. Places | Group × Object | 10 |  |
|  |  | 15 | 15 | fMRI | Decision task | Faces/Places | Controls > Panic disorder | 18 | - |
|  |  | 15 | 15 | fMRI | Decision task | Faces > Rest | Controls > Panic disorder | 14 | - |
|  |  | 15 | 15 | fMRI | Decision task | Places > Rest | Controls > Panic disorder | 9 | - |
|  |  | 15 | 15 | fMRI | Decision task | Places > Faces | Controls > Panic disorder | 5 | - |
| Pfleiderer | 2010 | 20 | 20 | fMRI | Acoustic stimulation | A1/A2/A3 | Stimulation × Group | 7 |  |
|  |  | 20 | 20 | fMRI | Acoustic stimulation | A1 > R1 | Panic disorder > Controls | 9 | + |
|  |  | 20 | 20 | fMRI | Acoustic stimulation | A3 > A2 | Panic disorder > Controls | 2 | + |
|  |  | 20 |  | fMRI | Acoustic stimulation | A3 > R3 | Positive correl. w/ HAMA | 9 | + |
| Reinecke | 2015 | 18 | 18 | fMRI | Emotion regulation ^E^ | Maintain/Reappraise > Baseline | Panic disorder > Controls | 16 | + |
|  |  | 18 | 18 | fMRI | Emotion regulation ^E^ | Maintain vs. Reappraise | Task × Group | 6 |  |
| Ridderbusch | 2019 | 13 | 17 | fMRI | Fear conditioning ^E, L^ | T1, Overall, AA/AG > GG | Controls > Patients | 10 | - |
|  |  | 13 | 17 | fMRI | Fear conditioning ^E, L^ | T1, Full acquisition, AA/AG > GG | Controls > Patients | 3 | - |
|  |  | 13 | 17 | fMRI | Fear conditioning ^E, L^ | T1, Full extinction, AA/AG > GG | Controls > Patients | 11 | - |
|  |  | 13 | 17 | fMRI | Fear conditioning ^E, L^ | T1, Overall, CS+ > CS-, AA/AG > GG | Patients > Controls | 1 | + |
|  |  | 13 | 17 | fMRI | Fear conditioning ^E, L^ | T1, Early acquisition, CS+ > CS-, AA/AG > GG | Patients > Controls | 10 | + |
|  |  | 13 | 17 | fMRI | Fear conditioning ^E, L^ | T1, Late acquisition, CS+ < CS-, AA/AG > GG | Patients > Controls | 4 | + |
|  |  | 13 | 17 | fMRI | Fear conditioning ^E, L^ | T1, Full extinction, CS+ > CS-, AA/AG > GG | Patients > Controls | 2 | + |
| Ridderbusch | 2020 | 12 | 7 | fMRI | Fear conditioning ^E, L^ | Late acquisition, S/S > L/L | Patients > Controls | 2 | + |
|  |  | 12 | 7 | fMRI | Fear conditioning ^E, L^ | Early extinction, S/S > L/L | Controls > Patients | 11 | - |
|  |  | 12 | 7 | fMRI | Fear conditioning ^E, L^ | Late extinction, S/S > L/L | Patients > Controls | 4 | + |
|  |  | 12 | 7 | fMRI | Fear conditioning ^E, L^ | Early acquisition, CS+ < CS-, S/S > L/L | Patients > Controls | 3 | + |
|  |  | 12 | 7 | fMRI | Fear conditioning ^E, L^ | Late extinction, CS+ > CS-, S/S > L/L | Patients > Controls | 2 | + |
| Schwarzmeier | 2019 | 10 | 10 | fMRI | Fear conditioning ^E, L^ | A1/A2: CS+ > CS- | Panic disorder > Controls | 2 | + |
|  |  | 10 | 10 | fMRI | Fear conditioning ^E, L^ | A1: CS+ > CS- | Panic disorder > Controls | 5 | + |
|  |  | 10 | 10 | fMRI | Fear conditioning ^E, L^ | ER2: CS+ > CS- | Panic disorder > Controls | 1 | + |
|  |  | 10 | 10 | fMRI | Fear conditioning ^E, L^ | ER3: CS+ > CS- | Panic disorder > Controls | 2 | + |
|  |  | 10 | 10 | fMRI | Fear conditioning ^E, L^ | A1: CS+ > CS- | Controls > Panic disorder | 1 | - |
|  |  | 10 | 10 | fMRI | Fear conditioning ^E, L^ | A2: CS+ > CS- | Controls > Panic disorder | 6 | - |
|  |  | 10 | 10 | fMRI | Fear conditioning ^E, L^ | ET1/ET2/ET3: CS+ > CS- | Controls > Panic disorder | 3 | - |
|  |  | 10 | 10 | fMRI | Fear conditioning ^E, L^ | ET1: CS+ > CS- | Controls > Panic disorder | 4 | - |
|  |  | 10 | 10 | fMRI | Fear conditioning ^E, L^ | ER1: CS+ > CS- | Controls > Panic disorder | 2 | - |
| Straube | 2014 | 20 | 20 | fMRI | Fear conditioning ^E, L^ | CS+ vs. CS- | Group × Time | 1 |  |
| Wade-B. | 2020 | 20 | 22 | fMRI | Emotional face processing ^E^ | Face perception, Pos. correl. w/ absolute valence (unc.) | Controls > Panic disorder | 2 | - |
|  |  | 20 | 22 | fMRI | Emotional face processing ^E^ | Face perc., Pos. corr. w/ abs. val. (unc., cov. Gender) | Controls > Panic disorder | 3 | - |
| Wang | 2021 | 26 | 25 | fMRI | Emotion regulation ^E^ | Non-negative > Negative description | Controls > Panic disorder | 2 | - |
|  |  | 26 | 25 | fMRI | Emotion regulation ^E^ | Non-negative > Negative description | Panic disorder > Controls | 1 | + |
| Wintermann | 2013 | 13 | 13 | fMRI | Olfactory emotion induction ^E^ | Artificial > No odor | Controls > Panic disorder | 3 | - |
|  |  | 13 | 13 | fMRI | Olfactory emotion induction ^E^ | Ergometry > No odor | Panic disorder > Controls | 2 | + |
|  |  | 13 | 13 | fMRI | Olfactory emotion induction ^E^ | Ergometry > TSST | Panic disorder > Controls | 2 | + |
| Wittmann | 2018 | 51 | 51 | fMRI | Emotion induction ^E^ | Agoraphobia-specific > Neutral cue (T1) | Panic disorder > Controls | 15 | + |
|  |  | 51 | 51 | fMRI | Emotion induction ^E^ | Agoraphobia-specific > Neutral cue (T1) | Controls > Panic disorder | 3 | - |
|  |  | 51 | 51 | fMRI | Emotion induction ^E^ | Agoraphobia-specific > Neutral cue (T2) | Panic disorder > Controls | 3 | + |
|  |  | 51 | 51 | fMRI | Emotion induction ^E^ | Agoraphobia-specific > Neutral cue (T2) | Controls > Panic disorder | 3 | - |
|  |  | 51 | 51 | fMRI | Emotion induction ^E^ | Agoraphobia-specific > Neutral picture (T1) | Panic disorder > Controls | 21 | + |
|  |  | 51 | 51 | fMRI | Emotion induction ^E^ | Agoraphobia-specific > Neutral picture (T1) | Controls > Panic disorder | 26 | - |
|  |  | 51 | 51 | fMRI | Emotion induction ^E^ | Agoraphobia-specific > Neutral picture (T2) | Panic disorder > Controls | 5 | + |
|  |  | 51 | 51 | fMRI | Emotion induction ^E^ | Agoraphobia-specific > Neutral picture (T2) | Controls > Panic disorder | 13 | - |
| Yang | 2020 | 103 | 103 | fMRI | Emotion induction ^E^ | T-S > N-S words (T1) | Panic disorder > Controls | 9 | + |
|  |  | 39 | 39 | fMRI | Emotion induction ^E^ | T-S > N-S words (T2) | Panic disorder > Controls | 1 | + |
|  |  | 103 | 103 | fMRI | Emotion induction ^E^ | N-S > T-S words (T1) | Panic disorder > Controls | 17 | + |
|  |  | 39 | 39 | fMRI | Emotion induction ^E^ | N-S > T-S words (T2) | Panic disorder > Controls | 26 | + |

BAI, Beck Anxiety Inventory; ^E^, experiment involving emotional stimuli; HAMA, Hamilton Anxiety Scale; HC, healthy controls; fMRI, functional magnetic resonance imaging; ^L^, experiment involving learning (conditioning, extinction, or reconsolidation); PD, panic disorder; PET, positron emission tomography

**Supplementary Table 3**

Neurotransmitter receptors and transporters included in the analyses

| Receptor/  transporter | Neurotransmitter | Tracer | Measure | N (males) | Age | Reference |
| --- | --- | --- | --- | --- | --- | --- |
| 5-HT_1A_ | Serotonin | [^11^C]WAY-100635 | BPND | 35 (18) | 26.3±5.2 | Savli et al. 2012 |
| 5-HT_1B_ | Serotonin | [^11^C]AZ10419369 | Bmax | 36 (24) | 27.8±6.9 | Beliveau et al. 2017 |
| 5-HT_2A_ | Serotonin | [^18^F]Altanserin | BPND | 19 (11) | 28.2±5.7 | Savli et al. 2012 |
| 5-HT_4_ | Serotonin | [^11^C]SB207145 | Bmax | 59 (41) | 25.9±5.3 | Beliveau et al. 2017 |
| 5-HTT | Serotonin | [^11^C]DASB | BPND | 30 (19) | 30.5±9.5 | Savli et al. 2012 |
| D_1_ | Dopamine | [^11^C]233390 | BPND | 13 (6) | 33±13 | Kaller et al. 2017 |
| D_2_ | Dopamine | [11C]RAC | BPND | 7 (7) | 24.5±3.5 | Alakurtti et al. 2015 |
| DAT^T^ | Dopamine | [^123^I]-FP-CIT | SUVR | 174 (109) | 61±11 | Dukart et al. 2018 |
| GABA_A_ | GABA | [^11^C]Flumazenil | BPND | 6 (6) | 43±4 | Dukart et al. 2018 |
| κ-opioid | Opioid | [^11^C]LY2795050 | BPND | 10 (10) | 28.7±7.4 | Shokri-Koj. et al. 2022 |
| mGluR_5_ | Glutamate | [^11^C]ABP688 | BPND | 74 (25) | 20±3.0 | Smart et al. 2019 |
| µ-opioid | Opioid | [^11^C]Carfentanil | BPND | 204 (132) | 32.4±10.8 | Kantonen et al. 2020 |
| NAT | Noradrenaline | [^11^C]MRB | BPND | 10 (6) | 33.3±10.0 | Hesse et al. 2017 |
| NMDA | Glutamate | [^18^F]GE-179 | V_T_ | 29 (21) | 41±13 | Galovic et al. 2023 |
| VAChT^T^ | Acetylcholine | [^18^F]FEOBV | SUVR | 18(5) | 66.8±6.8 | Aghourian et al. 2017 |

BP_ND_, non-displaceable binding potential; B_max_, converted from binding potential using autoradiography-derived densities; SUVR, standard uptake value ratio; VT, tracer distribution volume. The superscript T indicates transporters.

**Supplementary References**

Aghourian M, Legault-Denis C, Soucy JP, Rosa-Neto P, Gauthier S, Kostikov A, Gravel P, Bédard MA. Quantification of brain cholinergic denervation in Alzheimer's disease using PET imaging with [18F]-FEOBV. Mol Psychiatry 2017;22:1531–8.

Alakurtti K, Aalto S, Johansson JJ, Någren K, Tuokkola T, Oikonen V, Laine M, Rinne JO. Reproducibility of striatal and thalamic dopamine D2 receptor binding using [11C]raclopride with high-resolution positron emission tomography. J Cereb Blood Flow Metab 2011;31:155–65.

Ball TM, Ramsawh HJ, Campbell-Sills L, Paulus MP, Stein MB. Prefrontal dysfunction during emotion regulation in generalized anxiety and panic disorders. Psychol Med 2013;43:1475–86.

Beliveau V, Ganz M, Feng L, Ozenne B, Højgaard L, Fisher PM, Svarer C, Greve DN, Knudsen GM. A high-resolution in vivo atlas of the human brain's serotonin system. J Neurosci 2017;37:120–28.

Boshuisen ML, Ter Horst GJ, Paans AM, Reinders AA, den Boer JA. rCBF differences between panic disorder patients and control subjects during anticipatory anxiety and rest. Biol Psychiatry 2002;52:126–35.

Chechko N, Wehrle R, Erhardt A, Holsboer F, Czisch M, Sämann PG. Unstable prefrontal response to emotional conflict and activation of lower limbic structures and brainstem in remitted panic disorder. PLoS One 2009;4:e5537.

Dresler T, Hindi Attar C, Spitzer C, Löwe B, Deckert J, Büchel C, Ehlis AC, Fallgatter AJ. Neural correlates of the emotional Stroop task in panic disorder patients: an event-related fMRI study. J Psychiatr Res 2012;46:1627–34.

Dukart J, Holiga Š, Chatham C, Hawkins P, Forsyth A, McMillan R, Myers J, Lingford-Hughes AR, Nutt DJ, Merlo-Pich E, Risterucci C, Boak L, Umbricht D, Schobel S, Liu T, Mehta MA, Zelaya FO, Williams SC, Brown G, Paulus M, Honey GD, Muthukumaraswamy S, Hipp J, Bertolino A, Sambataro F. Cerebral blood flow predicts differential neurotransmitter activity. Sci Rep 2018;8:4074.

Eickhoff SB, Bzdok D, Laird AR, Kurth F, Fox PT. Activation likelihood estimation meta-analysis revisited. Neuroimage 2012;59:2349–61.

Engel KR, Obst K, Bandelow B, Dechent P, Gruber O, Zerr I, Ulrich K, Wedekind D. Functional MRI activation in response to panic-specific, non-panic aversive, and neutral pictures in patients with panic disorder and healthy controls. Eur Arch Psychiatry Clin Neurosci. 2016;266:557–66.

Feldker K, Heitmann CY, Neumeister P, Bruchmann M, Vibrans L, Zwitserlood P, Straube T. Brain responses to disorder-related visual threat in panic disorder. Hum Brain Mapp 2016;37:4439–53.

Feldker K, Heitmann CY, Neumeister P, Brinkmann L, Bruchmann M, Zwitserlood P, Straube T. Cardiorespiratory concerns shape brain responses during automatic panic-related scene processing in patients with panic disorder. J Psychiatry Neurosci 2018;43:26–36.

Fonzo GA, Ramsawh HJ, Flagan TM, Sullivan SG, Letamendi A, Simmons AN, Paulus MP, Stein MB. Common and disorder-specific neural responses to emotional faces in generalised anxiety, social anxiety and panic disorders. Br J Psychiatry 2015;206:206–15.

Galovic M, Al-Diwani A, Vivekananda U, Walker MC, Irani SR, Koepp MJ; NEST Investigators. In vivo N-methyl-d-aspartate receptor (NMDAR) density as assessed using positron emission tomography during recovery from NMDAR-antibody encephalitis. JAMA Neurol 2023;80:211–3.

Gechter J, Liebscher C, Geiger MJ, Wittmann A, Schlagenhauf F, Lueken U, Wittchen HU, Pfleiderer B, Arolt V, Kircher T, Straube B, Deckert J, Weber H, Herrmann MJ, Reif A, Domschke K, Ströhle A. Association of NPSR1 gene variation and neural activity in patients with panic disorder and agoraphobia and healthy controls. Neuroimage Clin 2019;24:102029.

Gorka SM, Huggins AA, Fitzgerald DA, Nelson BD, Phan KL, Shankman SA. Neural response to reward anticipation in those with depression with and without panic disorder. J Affect Disord 2014;164:50–6.

Held-Poschardt D, Sterzer P, Schlagenhauf F, Pehrs C, Wittmann A, Stoy M, Hägele C, Knutson B, Heinz A, Ströhle A. Reward and loss anticipation in panic disorder: An fMRI study. Psychiatry Res Neuroimaging 2018;271:111–7.

Hesse S, Becker GA, Rullmann M, Bresch A, Luthardt J, Hankir MK, Zientek F, Reißig G, Patt M, Arelin K, Lobsien D, Müller U, Baldofski S, Meyer PM, Blüher M, Fasshauer M, Fenske WK, Stumvoll M, Hilbert A, Ding YS, Sabri O. Central noradrenaline transporter availability in highly obese, non-depressed individuals. Eur J Nucl Med Mol Imaging 2017;44:1056–64.

Hope ACA. A simplified Monte Carlo significance test procedure. J R Stat Soc Series B Stat 1968;30:582–98.

van den Heuvel OA, Mataix-Cols D, Zwitser G, Cath DC, van der Werf YD, Groenewegen HJ, van Balkom AJ, Veltman DJ. Common limbic and frontal-striatal disturbances in patients with obsessive compulsive disorder, panic disorder and hypochondriasis. Psychol Med 2011;41:2399–410.

Jin H, Zhang B, Cui H, Li W, Li H, Hu Q, Wang J, Li C. Altered function of superior parietal lobule associated with perceptive awareness in first-episode drug-naïve panic disorders: A preliminary fMRI study. Neuropsychiatr Dis Treat 2020;16:1653–59.

Kaldewaij R, Reinecke A, Harmer CJ. A lack of differentiation in amygdala responses to fearful expression intensity in panic disorder patients. Psychiatry Res Neuroimaging 2019;291:18–25.

Kaller S, Rullmann M, Patt M, Becker GA, Luthardt J, Girbardt J, Meyer PM, Werner P, Barthel H, Bresch A, Fritz TH, Hesse S, Sabri O. Test-retest measurements of dopamine D1-type receptors using simultaneous PET/MRI imaging. Eur J Nucl Med Mol Imaging 2017;44:1025–32.

Kantonen T, Karjalainen T, Isojärvi J, Nuutila P, Tuisku J, Rinne J, Hietala J, Kaasinen V, Kalliokoski K, Scheinin H, Hirvonen J, Vehtari A, Nummenmaa L. Interindividual variability and lateralization of μ-opioid receptors in the human brain. Neuroimage 2020:217:116922.

Kircher T, Arolt V, Jansen A, Pyka M, Reinhardt I, Kellermann T, Konrad C, Lueken U, Gloster AT, Gerlach AL, Ströhle A, Wittmann A, Pfleiderer B, Wittchen HU, Straube B. Effect of cognitive-behavioral therapy on neural correlates of fear conditioning in panic disorder. Biol Psychiatry 2013;73:93–101.

Korgaonkar MS, Tran J, Felmingham KL, Williams LM, Bryant RA. Neural correlates of emotional processing in panic disorder. Neuroimage Clin 2021;32:102902.

Liebscher C, Wittmann A, Gechter J, Schlagenhauf F, Lueken U, Plag J, Straube B, Pfleiderer B, Fehm L, Gerlach AL, Kircher T, Fydrich T, Deckert J, Wittchen HU, Heinz A, Arolt V, Ströhle A. Facing the fear--clinical and neural effects of cognitive behavioural and pharmacotherapy in panic disorder with agoraphobia. Eur Neuropsychopharmacol 2016;26:431–44.

Lueken U, Straube B, Reinhardt I, Maslowski NI, Wittchen HU, Ströhle A, Wittmann A, Pfleiderer B, Konrad C, Ewert A, Uhlmann C, Arolt V, Jansen A, Kircher T. Altered top-down and bottom-up processing of fear conditioning in panic disorder with agoraphobia. Psychol Med 2014;44:381–94.

Maddock RJ, Buonocore MH, Kile SJ, Garrett AS. Brain regions showing increased activation by threat-related words in panic disorder. Neuroreport 2003;14:325–8.

Magnotta VA, Johnson CP, Follmer R, Wemmie JA. Functional t1ρ imaging in panic disorder. Biol Psychiatry 2014;75:884–91.

McIntosh RC, Hoshi RA, Timpano KR. Take my breath away: Neural activation at breath-hold differentiates individuals with panic disorder from healthy controls. Respir Physiol Neurobiol 2020;277:103427.

Meyer JH, Swinson R, Kennedy SH, Houle S, Brown GM. Increased left posterior parietal-temporal cortex activation after D-fenfluramine in women with panic disorder. Psychiatry Res 2000;98:133–43.

Petrowski K, Wintermann G, Smolka MN, Huebner T, Donix M. The neural representation of emotionally neutral faces and places in patients with panic disorder with agoraphobia. J Affect Disord 2014;152–154:454–61.

Pfleiderer B, Zinkirciran S, Michael N, Hohoff C, Kersting A, Arolt V, Deckert J, Domschke K. Altered auditory processing in patients with panic disorder: a pilot study. World J Biol Psychiatry 2010;11:945–55.

Reinecke A, Filippini N, Berna C, Western DG, Hanson B, Cooper MJ, Taggart P, Harmer CJ. Effective emotion regulation strategies improve fMRI and ECG markers of psychopathology in panic disorder: Implications for psychological treatment action. Transl Psychiatry 2015;5:e673.

Ridderbusch IC, Richter J, Yang Y, Hoefler M, Weber H, Reif A, Hamm A, Pané-Farré CA, Gerlach AL, Stroehle A, Pfleiderer B, Arolt V, Wittchen HU, Gloster A, Lang T, Helbig-Lang S, Fehm L, Pauli P, Kircher T, Lueken U, Straube B. Association of rs7688285 allelic variation coding for GLRB with fear reactivity and exposure-based therapy in patients with panic disorder and agoraphobia. Eur Neuropsychopharmacol 2019;29:1138–51.

Ridderbusch IC, Yang Y, Weber H, Reif A, Herterich S, Ströhle A, Pfleiderer B, Arolt V, Wittchen HU, Lueken U, Kircher T, Straube B. Neural correlates of NOS1 ex1f-VNTR allelic variation in panic disorder and agoraphobia during fear conditioning and extinction in fMRI. Neuroimage Clin 2020;27:102268.

Savli M, Bauer A, Mitterhauser M, Ding YS, Hahn A, Kroll T, Neumeister A, Haeusler D, Ungersboeck J, Henry S, Isfahani SA, Rattay F, Wadsak W, Kasper S, Lanzenberger R. Normative database of the serotonergic system in healthy subjects using multi-tracer PET. Neuroimage 2012;63:447–59.

Shokri-Kojori E, Naganawa M, Ramchandani VA, Wong DF, Wang GJ, Volkow ND. Brain opioid segments and striatal patterns of dopamine release induced by naloxone and morphine. Hum Brain Mapp 2022;43:1419–30.

Smart K, Cox SML, Scala SG, Tippler M, Jaworska N, Boivin M, Séguin JR, Benkelfat C, Leyton M. Sex differences in [11C]ABP688 binding: a positron emission tomography study of mGlu5 receptors. Eur J Nucl Med Mol Imaging 2019;46:1179–83.

Schwarzmeier H, Kleint NI, Wittchen HU, Ströhle A, Hamm AO, Lueken U. Characterizing the nature of emotional-associative learning deficits in panic disorder: An fMRI study on fear conditioning, extinction training and recall. Eur Neuropsychopharmacol 2019;29:306–318.

Straube B, Lueken U, Jansen A, Konrad C, Gloster AT, Gerlach AL, Ströhle A, Wittmann A, Pfleiderer B, Gauggel S, Wittchen U, Arolt V, Kircher T. Neural correlates of procedural variants in cognitive-behavioral therapy: A randomized, controlled multicenter fMRI study. Psychother Psychosom 2014;83:222–33.

Wade-Bohleber LM, Thoma R, Gerber AJ. Neural correlates of subjective arousal and valence in health and panic disorder. Psychiatry Res Neuroimaging 2020;305:111186.

Wang HY, Xu GQ, Ni MF, Zhang CH, Li XL, Chang Y, Sun XP, Zhang BW. Neural basis of implicit cognitive reappraisal in panic disorder: An event-related fMRI study. J Transl Med 2021;19:304.

Wintermann GB, Donix M, Joraschky P, Gerber J, Petrowski K. Altered olfactory processing of stress-related body odors and artificial odors in patients with panic disorder. PLoS One 2013;8:e74655.

Wittmann A, Schlagenhauf F, Guhn A, Lueken U, Elle M, Stoy M, Liebscher C, Bermpohl F, Fydrich T, Pfleiderer B, Bruhn H, Gerlach AL, Straube B, Wittchen HU, Arolt V, Heinz A, Kircher T, Ströhle A. Effects of cognitive behavioral therapy on neural processing of agoraphobia-specific stimuli in panic disorder and agoraphobia. Psychother Psychosom. 2018;87:350–65.

Yang Y, Lueken U, Richter J, Hamm A, Wittmann A, Konrad C, Ströhle A, Pfleiderer B, Herrmann MJ, Lang T, Lotze M, Deckert J, Arolt V, Wittchen HU, Straube B, Kircher T. Effect of CBT on biased semantic network in panic disorder: A multicenter fMRI study using semantic priming. Am J Psychiatry 2020;177:254–64.
